# Pyrazolopyrimide library screening in glioma cells discovers highly potent antiproliferative leads that target the PI3K/mTOR pathway

**DOI:** 10.1016/j.bmc.2019.115215

**Published:** 2020-01-01

**Authors:** Teresa Valero, Daniel J. Baillache, Craig Fraser, Samuel H. Myers, Asier Unciti-Broceta

**Affiliations:** Cancer Research UK Edinburgh Centre, MRC Institute of Genetics & Molecular Medicine, University of Edinburgh, Edinburgh EH4 2XR, UK

**Keywords:** Phenotypic screening, Ligand-based drug discovery, Kinase inhibitors, Pyrazolopyrimidines, Anticancer drugs

## Abstract

The search for novel targeted inhibitors active on glioblastoma multiforme is crucial to develop new treatments for this unmet clinical need. Herein, we report the results from a screening campaign against glioma cell lines using a proprietary library of 100 structurally-related pyrazolopyrimidines. Data analysis identified a family of compounds featuring a 2-amino-1,3-benzoxazole moiety **(eCF309** to **eCF334**) for their antiproliferative properties in the nM range. These results were validated in patient-derived glioma cells. Available kinase inhibition profile pointed to blockade of the PI3K/mTOR pathway as being responsible for the potent activity of the hits. Combination studies demonstrated synergistic activity by inhibiting both PI3Ks and mTOR with selective inhibitors. Based on the structure activity relationships identified in this study, five new derivatives were synthesized and tested, which exhibited potent activity against glioma cells but not superior to the dual PI3K/mTOR inhibitor and lead compound of the screening **eCF324**.

## Graphical abstract

**Figure:**
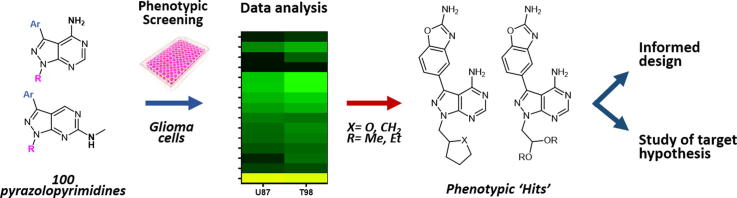

## 1 Introduction

Glioblastoma multiforme (GBM) is the most common and aggressive cancer that begins within the brain. It accounts for 45% of all primary brain tumors with an incidence of four to five per 100,000 adults per year in Europe.^1^ Without treatment, the median overall survival following diagnosis is merely 3 months, while with the best available surgical and adjuvant therapies (chemo and radiotherapy) can only be extended to 14–15 months.^2^ Despite a plethora of clinical trials across the world, GBM remains an unmet medical need, as novel strategies have failed to show an improvement over the standard of care, temozolomide (**TMZ**), an alkylating agent approved in the late 90s.^3^

Phenotypic screening campaigns are the major source of first-in-class drugs that eventually reach the clinic.^4^ In contrast to target-centric strategies, these cell-based compound screens survey changes in the cell phenotype, thereby embracing the complexity of the cell as a whole. This is especially important in cancer since redundancy, compensatory mechanisms, pathway cross-talks and plasticity are common and hardly predictable. In particular, GBM shows high heterogeneity at the molecular, genetic and epigenetic levels,^5^ which makes essential the use of models that recapitulate the disease, including the selection of various glioma cell types. Even if serendipitous, the discovery of hits through phenotypic screening on appropriate cell models can improve the odds of clinical translatability. For example, a phenotypic screening repurposing campaign in patient-derived glioma cells showed that combination of disulfiram (a drug used to treat alcoholism) and copper mediated promising activity and re-sensitization to **TMZ**, especially in glioma stem cell-like cells.^6^ This combination is currently in clinical phase.^7^

Phenotypic screening is typically followed by a target engagement/deconvolution step to identify the target/s and mechanism of action.^8^, ^9^ However, the appropriate target ID strategy must be optimized for each individual biological target and preclinical drug, representing a technically challenging step. In fact, some drug candidates reach regulatory approval without the actual knowledge of their action mechanism,^10^ which can potentially hinder further clinical development activities due to the lack of appropriate biomarkers.

**Fig. 1:**
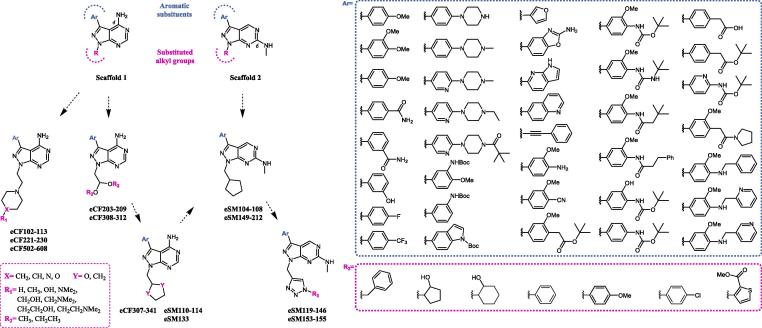
Chemical diversity and historic evolution of the pyrazolopyrimidines tested in this work. Library size = 100 compounds.

Using a pragmatic strategy that combines ligand-based drug design and phenotypic screening of selected cancer cell lines, our lab has generated several series of focused small molecule compounds featuring either a 4-amino or 6-methylamino pyrazolo[3,4-*d*]pyrimidine core (see Fig. 1) and discover potent “phenotypic” hits and lead compounds displaying a diversity of anticancer properties, including cell cycle arrest, pro-apoptotic and anti-migrative activities.^11^, ^12^, ^13^ Since these scaffolds are typically found in kinase inhibitors, kinome profiling of these hits and leads enabled fast elucidation of their target profile and the generation of structure activity relationship (SAR) to support subsequent optimization activities. Such campaigns resulted, for example, in the discovery of the potent SRC/nonABL kinase inhibitor eCF506,^11^ the selective mTOR inhibitor eCF309,^12^ or the potent FLT3/AXL/RET inhibitor eSM156.^13^

## 2 Results and discussion

A phenotypic screening campaign was performed using our in-house developed library^14^, ^15^ in search for small molecule inhibitors that could affect glioma cell proliferation. As shown in Fig. 1, the library used in the screening represents a highly-focused chemical-diversity space (see complete structural information in the Table 1 of the Suppl. Data). Importantly, this space is rich in bioactive compounds that have been shown to target a variety of protein, lipid and atypical kinases,^11^, ^12^, ^13^, ^14^, ^15^, ^16^, ^17^ thus improving the chances of finding active hits against glioma cells while facilitating the interpretation of potential SAR.

**Fig. 2:**
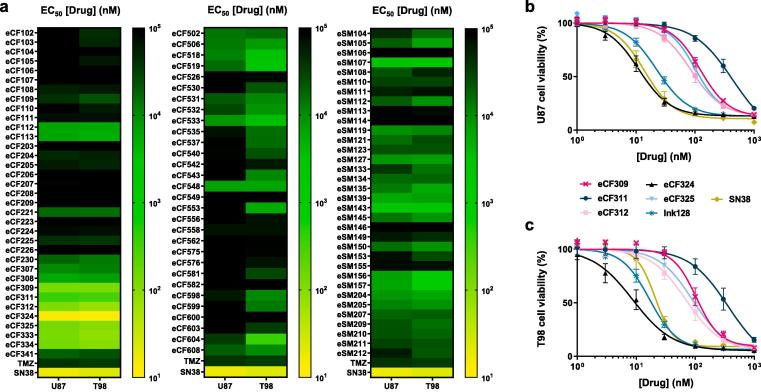
(a) Heatmaps of EC_50_ values of the 100 final compounds tested. Data represents mean values of three independent experiments in triplicates. (b and c) Dose response curves of most potent hits on (b) U87 and (c) T98 cells. Cells were treated with each inhibitor at concentrations ranging from 1 to 30,000 nM and PrestoBlue assay used at day 5 to detect viability. Data are means ± SEM of 3 independent experiments in triplicates.

The antiproliferative activity of a total of 100 compounds was tested against U87 and T98 glioma cell lines, using **TMZ**^3^ and the Topoisomerase I inhibitor **SN-38**^18^ as positive controls. Cells were treated with the library members for 5 d at three different concentrations (3, 30 and 300 µM) and cell viability determined using the PrestoBlue reagent. Half-maximal effective concentration (EC_50_) values were calculated from the corresponding dose-response curves and plotted in Fig.  2a as a heatmap to facilitate data analysis, with black meaning no activity and dark to light green to yellow transitions indicating decreasing EC_50_ values (=increasing potencies). Table 1 of the Suppl. Data contains the corresponding EC_50_ values ± SEM. Whereas compounds based on **scaffold 2** (third column, Fig.  2a) were found to be more active in general than those from **scaffold 1** (first and second columns, Fig.  2a), the highest activities were exhibited by a particular family of compounds from the **eCF** series (**eCF309** to **eCF334**). Structurally speaking, these potent compounds presented a 2-amino-1,3-benzoxazole moiety at the *C*3 position of the ring, a group that is also found in the mTOR inhibitor sapanisertib (a.k.a. **INK128**), a drug candidate currently in clinical development.^19^

To confirm the results, U87 and T98 cell viability assays were repeated for the most potent hits (**eCF309**, **eCF311**, **eCF312**, **eCF324**, **eCF325**, **eCF333** and **eCF334**, all of which feature a 2-amino-1,3-benzoxazole moiety at the *C*3 position) using a 7-point half-log dose–response study (1 to 1,000 nM). **INK128** and **SN-38** were used as positive controls. Resulting curves are shown in Fig.  2b and c. **eCF333** and **eCF334** (pure enantiomers of **eCF325**) presented identical dose response curves to that of **eCF325**, and thus their data were not included for clarity. As shown in Fig. 2b and c, **eCF324** was the most potent compound in both cell lines, displaying EC_50_ values of 13 and 10 nM in U87 and T98 cells, respectively; values that were superior to those of the positive controls **INK128** and **SN-38**. Of note, >200-fold activity gap was observed between lead compounds **eCF324** and **eCF311**; the latter presenting a methyl-1-3-dioxolane group at *N*1 instead of a methylcyclopentane. The rest of the hits exhibited EC_50_ values in the range of 100–150 nM. Structurally, these derivatives differed in the size of the alkyl group of the *N*1 position and the presence or absence of an oxygen atom in that moiety (see Suppl. Data). None of them exhibited superior potency than **eCF324**, indicating that the most lipophilic cyclopentylmethyl group found in this compound is optimal for activity.

**Fig. 3:**
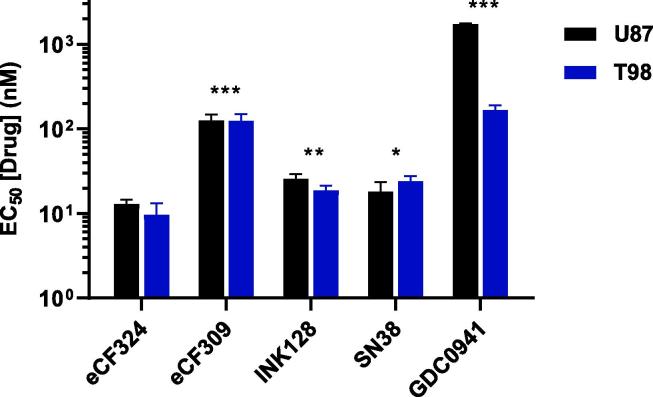
EC_50_ values for **eCF324**, **eCF309**, positive controls **INK128** and **SN-38** and pan-PI3K inhibitor **GDC0941** on glioma U87 and T98 cell lines. Cells were treated with each inhibitor at concentrations ranging from 1 to 1,000 nM and PrestoBlue assay used at day 5 to detect viability. Data are mean ± SEM of three independent experiments in triplicates, *P < 0.05, **P < 0.01, ***P < 0.001 compared to **eCF324**.

In order to correlate the antiproliferative activities of the phenotypic hits with potential target/s, we took advantage of kinase profiling data previously obtained for these compounds.^12^ As shown in the Suppl. Data, all the hits display high inhibitory activity for mTOR kinase, being **eCF309** the most selective (IC_50_ (mTOR) = 15 nM; IC_50_ (PI3Kα) = 981 nM) and **eCF324** the most potent (IC_50_ (mTOR) = 1.8 nM; IC_50_ (PI3Kα) = 3.5 nM). The antiproliferative potency exhibited by the selective mTOR inhibitor **eCF309** strongly supports the role of mTOR inhibition in the antiproliferative properties of the hits. On the other hand, the superior anti-glioma activity of **eCF324** suggests that its polypharmacological properties are beneficial for inhibiting glioma cell proliferation. To study the possible role of PI3K inhibition, additional cell viability studies were performed in U87 and T98 cells with the selective pan-PI3K inhibitor pictilisib (a.k.a. **GDC0941**^20^**)**. As shown in Fig. 3, although both **eCF309** and **eCF324** display superior activity than the pan-PI3K inhibitor **GDC0941**, this selective inhibitor exhibits sub-µM activity against T98 (equivalent to that of **eCF309**) and low µM activity in U87 cells.

**Fig. 4:**
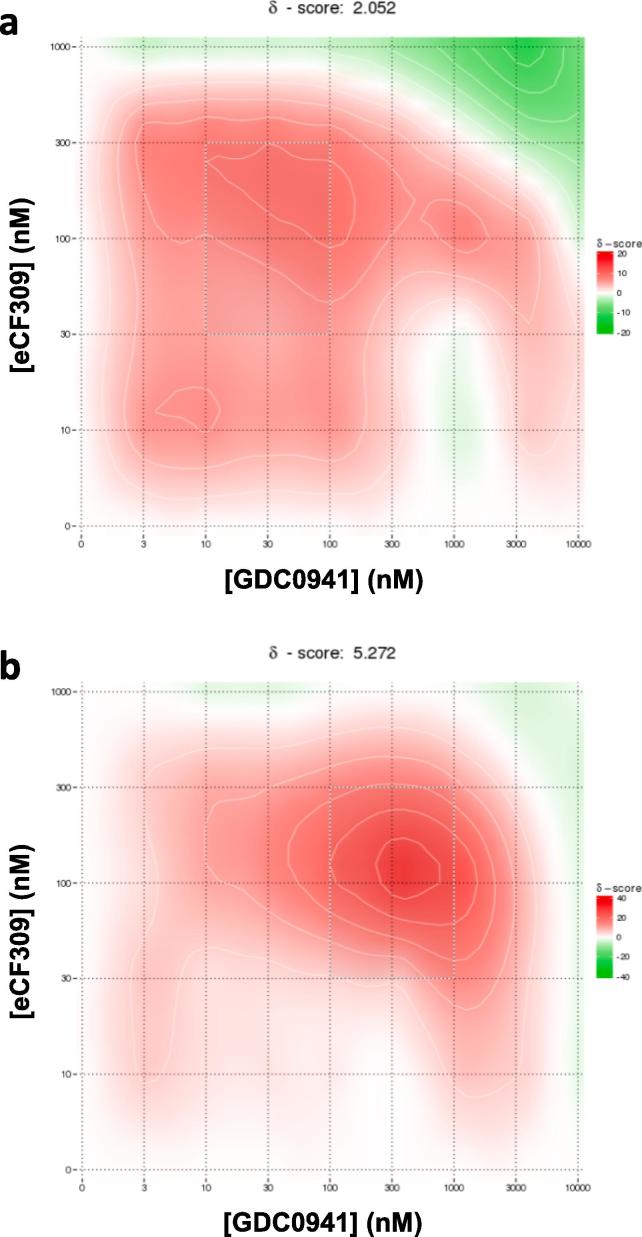
Synergy scores for the combinations of **eCF309** and **GDC0941** on (a) U87 and (b) T98 cells. Cells were treated with combinations of 1000, 300, 100, 30, 10, 0 nM of eCF309 and/or 10,000, 3000, 1000, 300, 100, 30, 10, 3, 0 nM of **GDC0941** during 5 days and PrestoBlue assay was used to detect viability. The most synergistic area is highlighted (white line). Data are ZIP synergy scores of means of three independent experiments.

Next, to test whether dual PI3K/mTOR inhibition could provide a therapeutic advantage to target glioma cells, cell viability studies were performed in U87 and T98 cells by combining **eCF309** with the pan-PI3K inhibitor **GDC0941** at a series of concentrations. Synergy scores for these combinations are presented in Fig. 4, with the red areas representing synergy and the green ones antagonism. Notably, strong synergistic activity was observed in most combinations, with maximum synergy found at 100 nM of **eCF309** and 300 nM of **GDC0941** in T98 cells. Since both compounds are known to be selective inhibitors of mTOR^12^ and PI3K,^20^ respectively, this study indicates that concurrent inhibition of mTOR and PI3Ks potentiate an antiproliferative effect in these two glioma cell lines. Combination doses for maximum synergy differed between U87 and T98 cells, suggesting different expression/activities of these oncogenic drivers in each cell line.

Combination experiments were also performed with **eCF324** and **GDC0941**, which also found synergistic activities (see Suppl. Data). This may indicate that the inherent inhibitory properties of **eCF324** on mTOR and PI3K are not optimally balanced to reach maximal synergistic anticancer activity and additional PI3K inhibition can promote further the antiproliferative effect mediated by this agent. Nevertheless, this possible interpretation needs to be taken with caution due to the known promiscuity of **eCF324**^12^; additional off-target effects may be at play in this case.

**Fig. 5:**
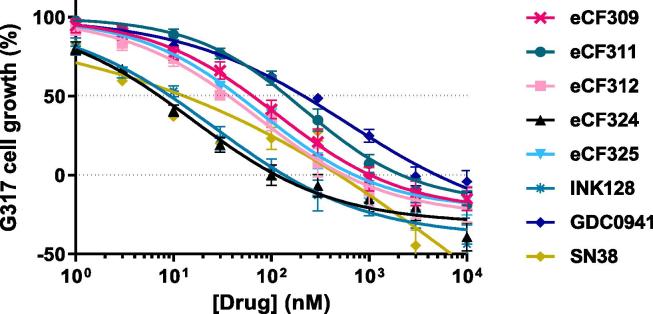
Dose response curves of most potent hits on the patient-derived cell line G317. Cells were treated with each inhibitor at concentrations ranging from 1 to 10,000 nM and PrestoBlue assay used at day 5 to detect viability. Cell growth was related to DMSO treatment (100%) and before drug treatment (0%), and GI_50_ values were extrapolated from the sigmoidal dose–response curve. Data are means ± SEM of 3 independent experiments in triplicates.

To corroborate the results in a more clinically-relevant model, a patient-derived GBM cell line, G317, was tested next. Fig. 5 shows the inhibition of cell growth by the most potent hits in this cell line. GI_50_ values (50% of growth inhibition) in G317 are represented in Table S1 (Suppl. Data). The choice of obtaining GI_50_ values instead of EC_50_ was made to determine if the compounds mediate cell growth arrest (cell viability values over the basal line) or cell death (cell viability values below the basal line). In agreement with previous results, compound **eCF324** exhibited the greatest potency with a GI_50_ valued of 7.2 nM, similar to **INK128** but higher than the Topoisomerase-I inhibitor **SN-38** and the pan-PI3K inhibitor **GDC0941**. Data from **eCF333** and **eCF334** showed identical dose response curves to **eCF325** and, therefore, were not shown in Fig. 5 for clarity. Of note, all compounds induced cell death, which contrast with the antiproliferative mode of action previously observed in breast and prostate cancer cells for these compounds.^12^ Notably, the selective mTOR inhibitor **eCF309** showed very potent activity in the patient derived cells, with a GI_50_ value of 65 nM, further evidence of the central role of mTOR in promoting cell growth and survival in GBM.^23^

**Fig. 6:**
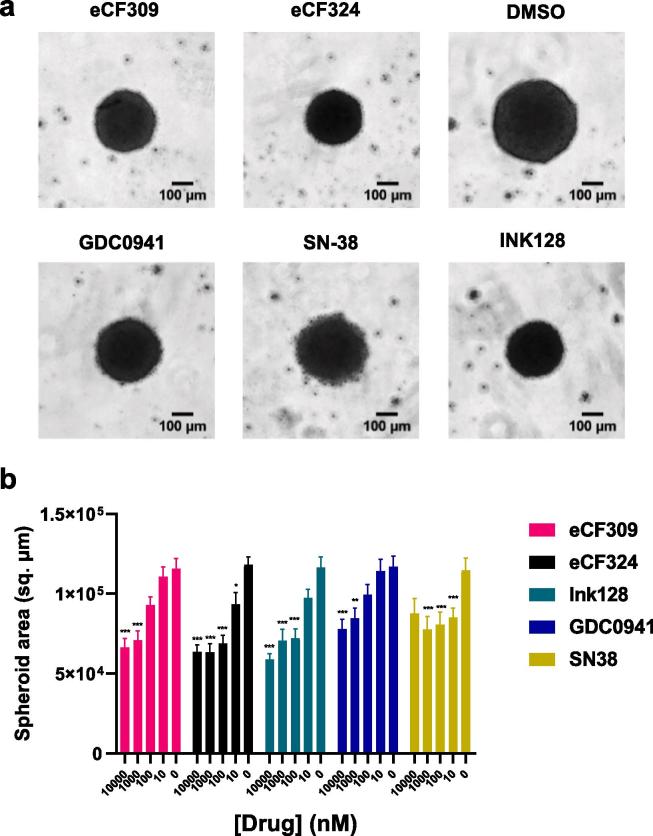
(a) Images of G317 spheroids on day 10. Spheroids were treated with 10 μM of each inhibitor during 8 days, images were acquired using ImageXpress. Scale bar = 100 μm. (b) Area of G317 patient derived spheroids at day 10. Spheroid area was calculated with a custom pipeline on CellProfiler. Data are mean ± SEM of 6 independent spheroids from two different cultures, ***P < 0.001, **P < 0.01 compared to DMSO controls (two-way ANOVA, Tukey post hoc test).

To test the compounds under conditions that better represent the tumour microenvironment, G317 cells were cultured in 3D and pre-formed spheroids treated with **eCF309**, **eCF324** and the positive controls at a range of concentrations (0.01–10 µM) for 8 days. As shown in Figure 6, inhibitors induced a dose dependent reduction of G317 spheroid size, with **eCF324** and **INK128** exhibiting the most potent antiproliferative activities.

Preliminary assessment of the safety profile of the hits was performed in two noncancerous cell lines: human brain vascular pericytes (HBVP) and mouse brain endothelial cells (bEnd3). Cell viability studies (see Suppl. Data) showed that the hits of the screening affected normal cell proliferation at concentrations above 3 µM, two to three orders of magnitude higher dose than that required to reduce proliferation of glioma cells.

**Fig. 7:**
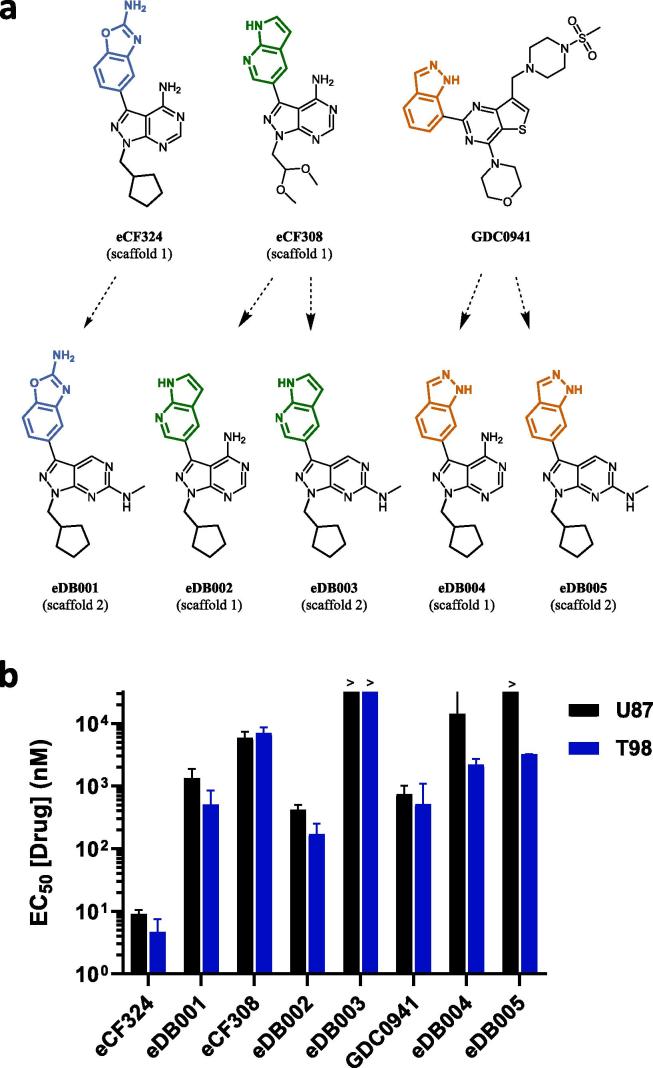
(a) New compounds synthesized. (b) EC_50_ values of novel compounds in U87 and T98 cell lines. Cells were treated with each inhibitor at concentrations ranging from 1 to 30,000 nM and PrestoBlue assay used at day 5 to detect viability. Data are means ± SEM of 3 independent experiments in triplicates.

Based on the lead compounds identified in the screening, five new derivatives were designed and synthesised to bring together the best structural features observed from the SAR of the screen. Compound **eDB001** was designed to incorporate the 2-amino-1,3-benzoxazole group found at C3 of **eCF324** into **scaffold 2**. The other four compounds incorporated the cyclopentylmethyl moiety found at the *N*1 position of **eCF324** and either an azaindole or indazole group at *C*3 in each scaffold to explore SAR further. **eDB002** and **eDB003** displayed the same group than **eSM145**, also found in **KW-2449**,^21^ a potent multi-kinase inhibitor of FLT3, ABL, and related kinases. An indazole group was used for **eDB004** and **eDB005** to mimic the structural properties of the selective pan-PI3K inhibitor **GDC0941**. As shown in Fig. 7, **eDB001** and **eDB002** displayed high activity in both cell lines, although their EC_50_ values did not reach the potency of **eCF324**. Given the results of the new derivatives, future optimization strategies should focus on **scaffold 1** and modify the 2-amino-1,3-benzoxazole moiety at *C*3 and the lipophilic group at *N*1 to enhance activity.

## 3 Materials and methods

### 3.1 General

Commercially available chemicals and anhydrous solvents were purchased from a range of suppliers, including: Acros Organics, Alfa Aesar, Fisher Scientific, Fluorochem, Matrix Scientific, Sigma Aldrich and VWR International. Cell culture plasticware was obtained from Greiner or Corning/Costar.

### 3.2 Cell culture

U87 and T98 cells (ATCC) were cultured in DMEM (Gibco), supplemented with 10% (v/v) FBS (Gibco) and 2 mM l-Glutamine (Gibco) in a standard incubator (95% humidity, 5% CO_2_), and subcultured twice per week through trypsinization. G317 patient-derived glioma cells were established by the Glioma Cellular Genetics Resource (gcgr.org.uk) funded by Cancer Research UK. Ethics approval for use of patient-derived cells and early access confirmation to reagents from the Glioma Cellular Genetics Resource assured that all ethical issues according to UK legislation are fulfilled. G317 were cultured in DMEM/HAMS-F12 (Sigma) supplemented with 1.5 g L^−1^ D-(+)-Glucose (Sigma), MEM non-essential aminoacids (Gibco), 0.015% (w/v) Penicillin-Streptomycin (Gibco), 0.012% (w/v) BSA (Gibco), 0.1 mM β-mercaptoethanol (Gibco), B27 (Gibco) and N2 supplements (Gibco). Cells were detached using accutase solution (Sigma) and subcultured once per week. Before adding to the cells, medium was supplemented with 10 ng ml^−1^ mouse EGF (Preprotech), 10 ng ml^−1^ human FGF (Preprotech), and 1 µg ml^−1^ Laminin (Sigma).

### 3.3 Cell viability assay

1 × 10^3^ U87 cells/well, 5 × 10^2^ T98 cells/well, 1 × 10^4^ G317 cells/well, 1 × 10^4^ HBVP cells/well and 1 × 10^4^ bEnd3 cells/well were seeded in 96-well plates. After 48 h in culture, cells were treated with the inhibitors for 5 d. For GI_50_ determination, the viability of a triplicate sample of each experiment before treatment was measured with the PrestoBlue Reagent (Invitrogen), to establish a basepoint 0% of growth. After 5 d, the viability was measured with PrestoBlue and related to 0% (indicating total cell death for EC_50_ determination or growth inhibition for GI_50_) and 100% viability (cells in the absence of drug). ZIP Synergy scores were obtained with Synergy finder.^22^ Viability percentage, EC_50_ and GI_50_ values were obtained using GraphPad Prism version 8.0.0 for Windows, GraphPad Software, San Diego, California USA, www.graphpad.com.

### 3.4 Spheroids assay

4 × 10^3^ G317 cells were seeded in an ultra-low attachment round bottom 96-well plate, centrifuged at 1000*g*, 4 °C for 10 min and allowed to form spheroids for 2 days. Spheroids were then incubated with the inhibitors at 4 concentrations ranging from 10 nM to 10 µM. Spheroids were imaged at day 10 using ImageXpress (Molecular Devices LLC) and processed with Image*J*.^25^ Spheroid area was calculated with a custom pipeline on CellProfiler.^26^

## 4 Conclusions

A series of structurally related pyrazolopyrimidine derivatives have been identified as potent inhibitors of glioma cell proliferation by a cell-based screening of a focused library of 100 pyrazolopyrimidines. The most potent hits displayed sub-micromolar activity against U87 and T98 glioma cell lines. The hits belonged to a group of compounds known to target mTOR and/or PI3Ks, including the selective mTOR inhibitor **eCF309** and the dual PI3K/mTOR inhibitor **eCF324**. Based on SAR identified in this study, new derivatives were designed, which exhibited potent activities against glioma cells but not superior to the lead compound **eCF324**. Synergy studies with the commercially-available selective pan-PI3K inhibitor **GDC0941** provided evidence of the synergistic effect of concurrently inhibiting mTOR and PI3Ks in glioma cell growth. The most potent hits were also validated in a patient-derived glioma cell model in 2D and 3D. This work further supports the search for inhibitors that can target PI3Ks and/or mTOR kinases^23^, ^24^ to fight GBM.

## Declaration of Competing Interest

The authors declare that they have no known competing financial interests or personal relationships that could have appeared to influence the work reported in this paper.
