# Supplementary material for: Pyrazolopyrimide library screening in glioma cells discovers highly potent antiproliferative leads that target the PI3K/mTOR pathway
Source: Bioorg Med Chem. 2020 Jan 1;28(1):115215. doi: 10.1016/j.bmc.2019.115215 (PMC6961122; doi:10.1016/j.bmc.2019.115215)
Supplement: Supplementary Data 1 [file mmc1.pdf]

# SUPPLEMENTARY DATA

# Pyrazolopyrimide library screening in glioma cells discovers highly potent antiproliferative leads that target the PI3K/mTOR pathway

Teresa Valero, Daniel J. Baillache, Craig Fraser, Samuel H. Myers and Asier Unciti-Broceta*

*Cancer Research UK Edinburgh Centre, MRC Institute of Genetics & Molecular Medicine*
*University of Edinburgh, Edinburgh EH4 2XR, UK.*

## Table of Contents

1. Structure of compounds tested and corresponding $EC_{50}$ values against glioma cells ............................. 2

1.1. Structures of positive controls TMZ, INK128 and SN-38 ............................................................. 21

2. IC50 values of most potent hits against a panel of recombinant kinases ............................................ 21

3. Synergy growth inhibition and ZIP synergy scores ........................................................................... 22

4. $GI_{50}$ values of most potent inhibitors on patient-derived cells ............................................................ 24

5. 3D cell proliferation assay ........................................................................................................... 25

6. $GI_{50}$ values of most potent inhibitors on glioma vs. noncancerous brain cells ...................................... 26

7. General chemistry methods ......................................................................................................... 27

8. Structures of new derivatives synthesized in this work ........................................................................ 28

9. Synthesis of new derivatives ......................................................................................................... 28

* Asier Unciti-Broceta. Tel.: +44-0131-651-8702.; e-mail: Asier.Unciti-Broceta@igmm.ed.ac.uk

## 1. Structure of compounds tested and corresponding $EC_{50}$ values against glioma cells

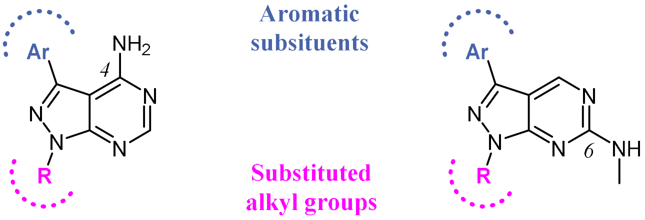


Scaffold 1 Scaffold 2

| Compound | Scaffold | Ar | R | $EC_{50}$ U87 ± SEM (nM) | $EC_{50}$ T98 ± SEM (nM) |
|---|---|---|---|---|---|
| eCF102 | 1 | 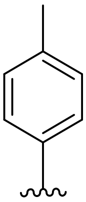 | 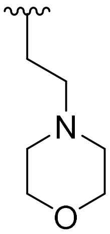  | >30000 | >30000 |
| eCF103 | 1 | 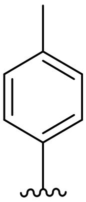 | 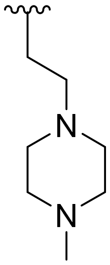  | >30000 | >30000 |
| eCF104 | 1 | 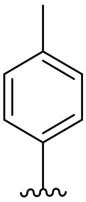 | 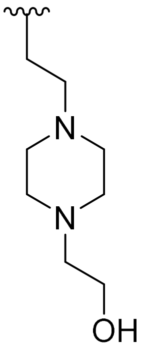  | >30000 | >30000 |

| | | | | | |
|---|---|---|---|---|---|
| eCF105 | 1 | 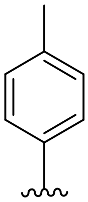 | 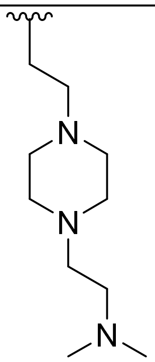 | >30000 | >30000 |
| eCF106 | 1 | 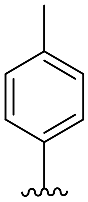 | 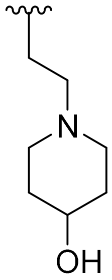 | >30000 | >30000 |
| eCF107 | 1 | 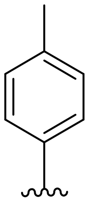 | 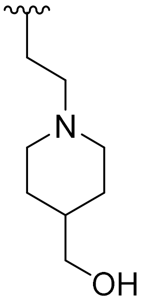 | >30000 | >30000 |
| eCF108 | 1 | 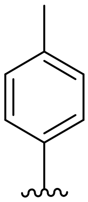 | 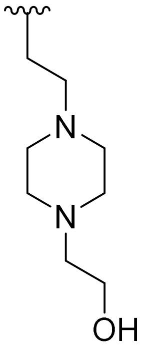 | >30000 | >30000 |
| eCF109 | 1 | 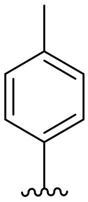 | 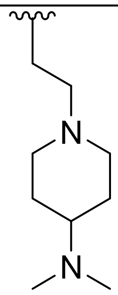 | >30000 | 26305±7298 |

| | | | | | |
|---|---|---|---|---|---|
| eCF110 | 1 | 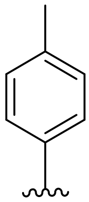 | 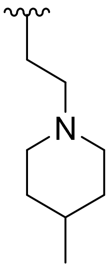 | >30000 | >30000 |
| eCF111 | 1 | 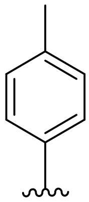 | 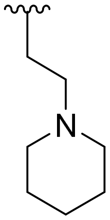 | >30000 | >30000 |
| eCF112 | 1 | 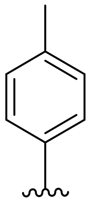 | 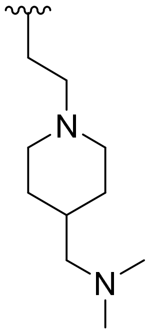 | 6634±1438 | 6283±1907 |
| eCF113 | 1 | 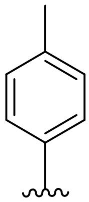 | 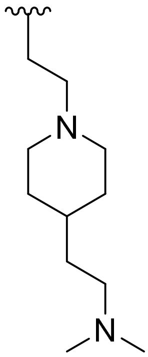 | 6739±1482 | 5724±1950 |
| eCF203 | 1 | 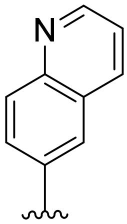 | 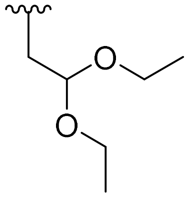 | >30000 | >30000 |

| | | | | | |
|---|---|---|---|---|---|
| eCF204 | 1 | 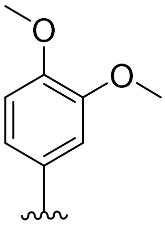 | 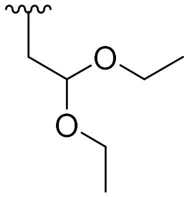 | >30000 | >30000 |
| eCF205 | 1 | 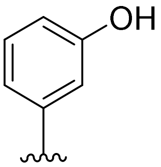 | 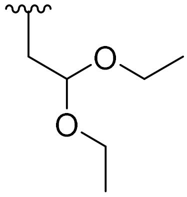 | >30000 | >30000 |
| eCF206 | 1 | 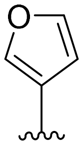 | 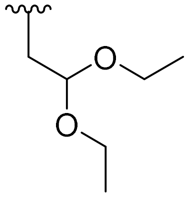 | >30000 | >30000 |
| eCF207 | 1 | 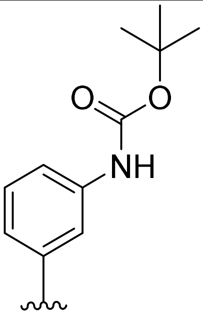 | 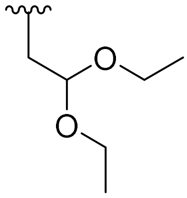 | >30000 | >30000 |
| eCF208 | 1 | 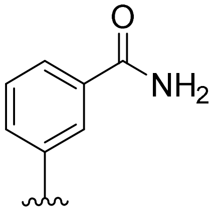 | 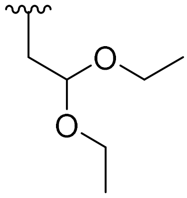 | >30000 | >30000 |
| eCF209 | 1 | 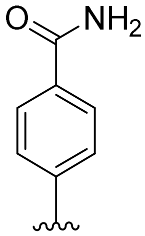 | 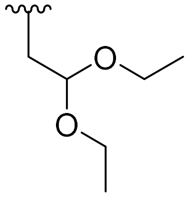 | >30000 | >30000 |

| | | | | | |
|---|---|---|---|---|---|
| eCF221 | 1 | 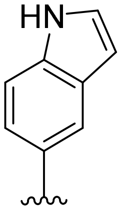 | 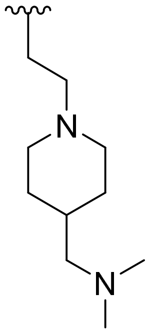 | 18685±4907 | 16811±3664 |
| eCF223 | 1 | 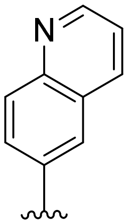 | 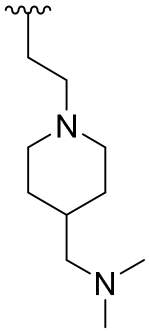 | >30000 | >30000 |
| eCF224 | 1 | 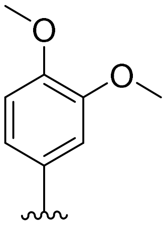 | 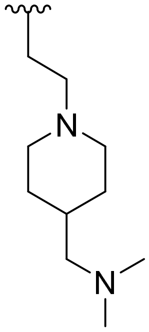 | >30000 | >30000 |
| eCF225 | 1 | 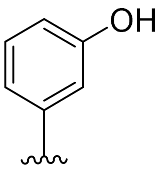 | 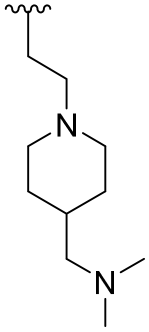 | >30000 | >30000 |

| | | | | | |
|---|---|---|---|---|---|
| eCF226 | 1 | 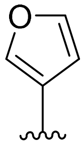 | 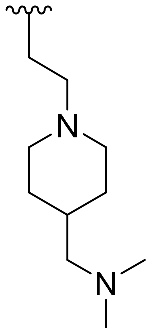 | >30000 | >30000 |
| eCF230 | 1 | 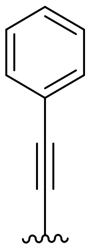 | 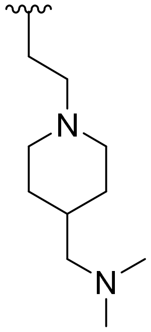 | 18245±1201 | 13684±1843 |
| eCF307 | 1 | 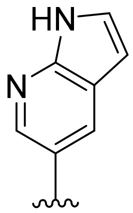 | 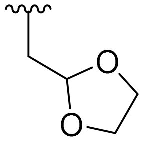 | 11262±2565 | 13128±2649 |
| eCF308 | 1 | 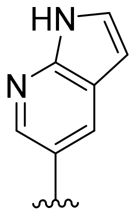 | 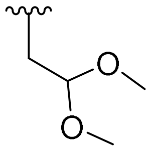 | 6162±1218 | 7338±1558 |
| eCF309 | 1 | 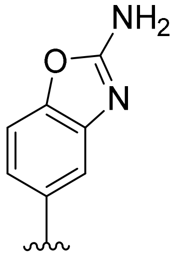 | 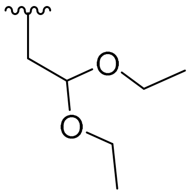 | 129±19 | 128±22 |
| eCF311 | 1 | 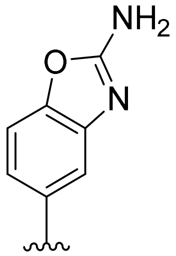 | 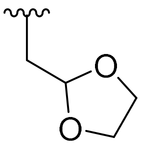 | 392±58 | 323±87 |

| | | | | | |
|---|---|---|---|---|---|
| eCF312 | 1 | 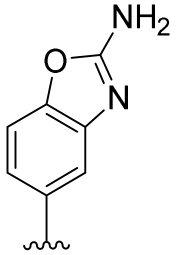 | 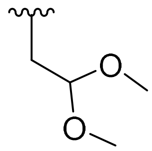 | 116±21 | 80±21 |
| eCF324 | 1 | 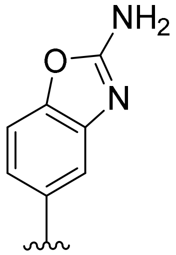 | 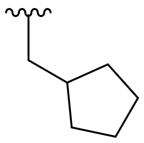 | 16±3 | 11±3 |
| eCF325 | 1 | 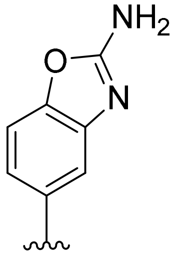 | 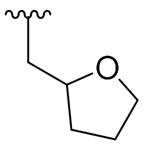 | 138±23 | 105±23 |
| eCF333 | 1 | 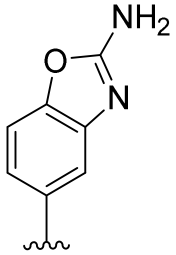 | 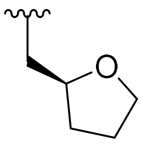 | 131±10 | 88±14 |
| eCF334 | 1 | 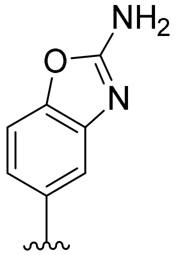 | 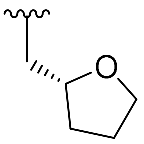 | 138±13 | 100±18 |
| eCF341 | 1 | 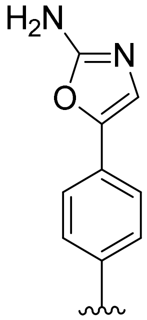 | 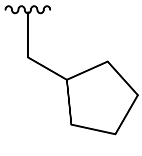 | 20338±2631 | 26190± **6565** |

| | | | | | |
|---|---|---|---|---|---|
| eCF502 | 1 | 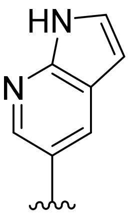 | 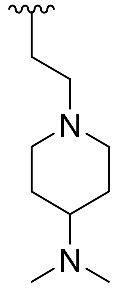 | 15230±2361 | 17571±3934 |
| eCF506 | 1 | 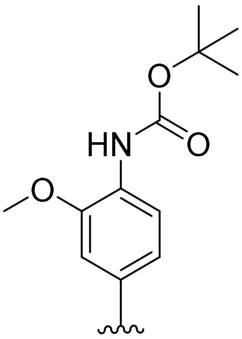 | 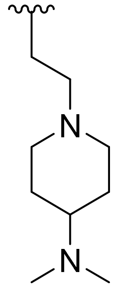 | 13644±809 | 9966±4884 |
| eCF518 | 1 | 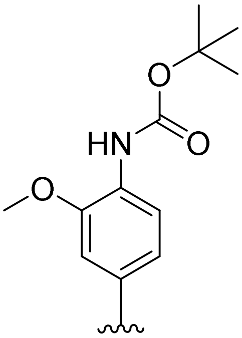 | 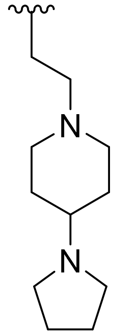 | 12021±168 | 3475±567 |
| eCF519 | 1 | 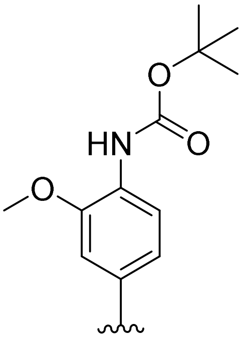 | 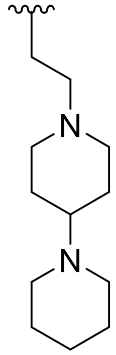 | 17035±2372 | 3142±331 |
| eCF526 | 1 | 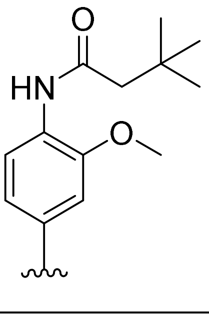 | 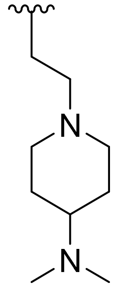 | >30000 | >30000 |

| | | | | | |
|---|---|---|---|---|---|
| eCF530 | 1 | 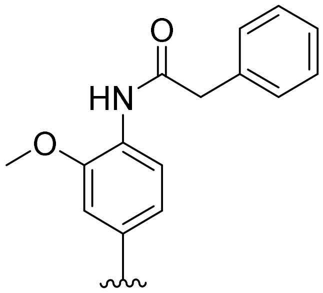 | 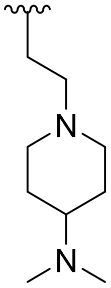 | >30000 | 22814±3210 |
| eCF531 | 1 | 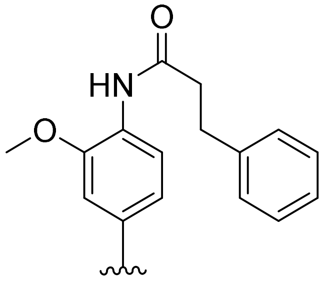 | 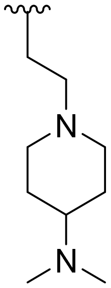 | 22606±5376 | 11141±1102 |
| eCF532 | 1 | 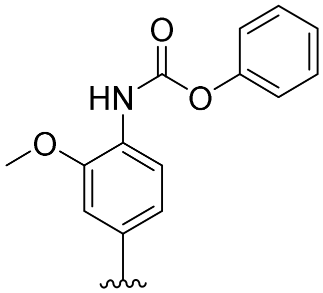 | 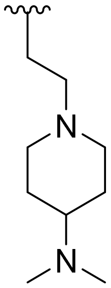 | 29163±3875 | 9099±1374 |
| eCF533 | 1 | 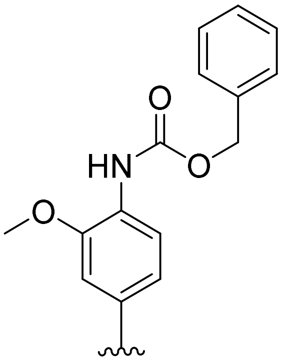 | 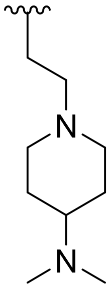 | 8224±671 | 3786±519 |
| eCF535 | 1 | 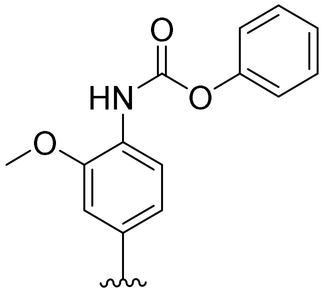 | 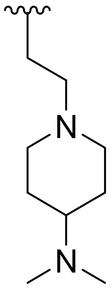 | >30000 | 16712±2802 |

| | | | | | |
|---|---|---|---|---|---|
| eCF537 | 1 | 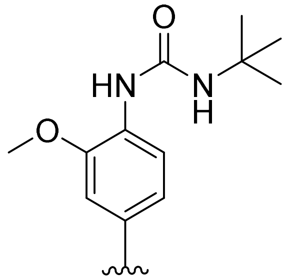 | 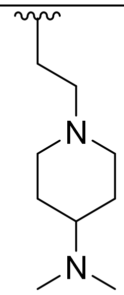 | >30000 | 17605±3242 |
| eCF540 | 1 | 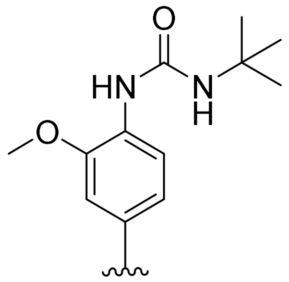 | 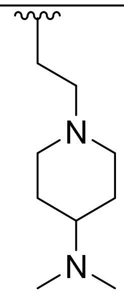 | >30000 | 24815±2680 |
| eCF542 | 1 | 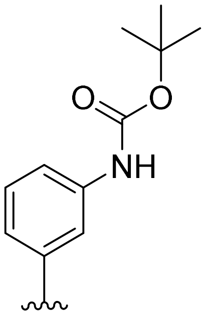 | 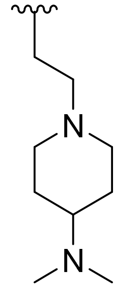 | >30000 | >30000 |
| eCF543 | 1 | 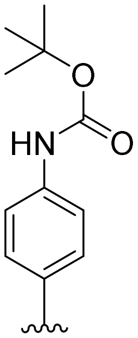 | 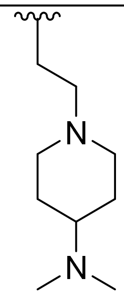 | >30000 | 11383±1444 |
| eCF548 | 1 | 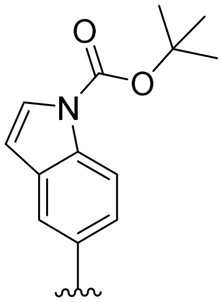 | 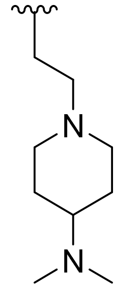 | 7182±1498 | 6868±1765 |

| | | | | | |
|---|---|---|---|---|---|
| eCF549 | 1 | 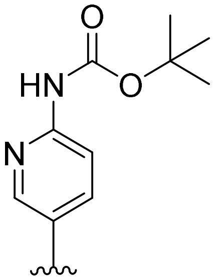 | 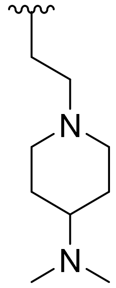 | >30000 | >30000 |
| eCF553 | 1 | 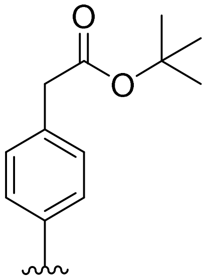 | 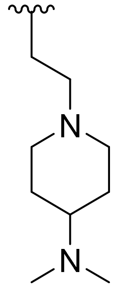 | >30000 | 6579±2181 |
| eCF556 | 1 | 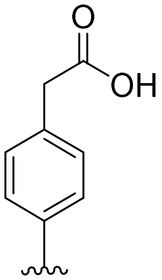 | 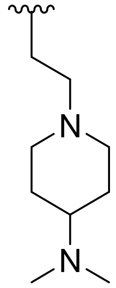 | >30000 | >30000 |
| eCF558 | 1 | 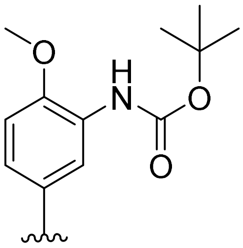 | 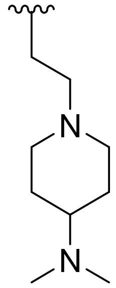 | >30000 | >30000 |
| eCF562 | 1 | 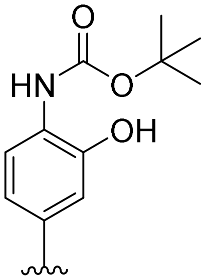 | 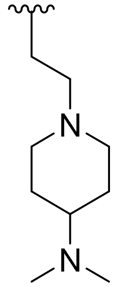 | >30000 | >30000 |

| | | | | | |
|---|---|---|---|---|---|
| eCF575 | 1 | 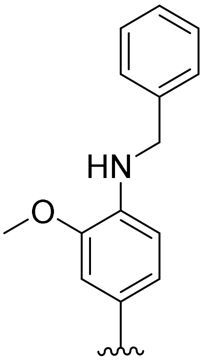 | 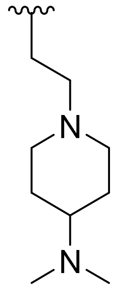 | >30000 | >30000 |
| eCF576 | 1 | 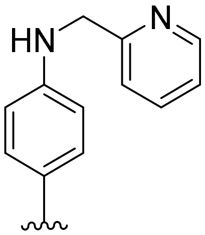 | 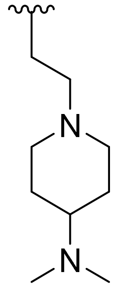 | >30000 | >30000 |
| eCF581 | 1 | 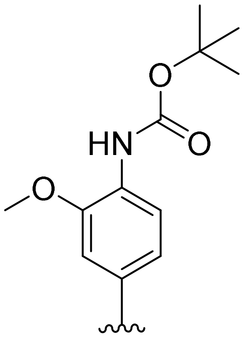 | 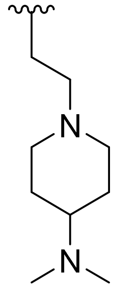 | >30000 | >30000 |
| eCF582 | 1 | 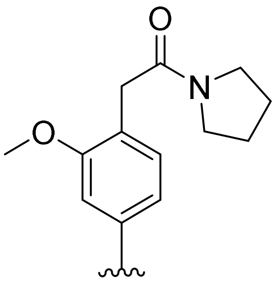 | 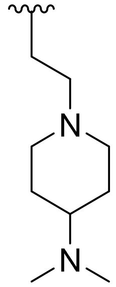 | >30000 | >30000 |
| eCF598 | 1 | 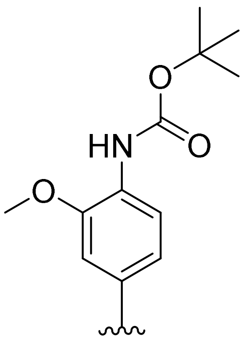 | 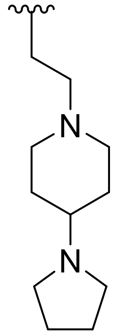 | >30000 | 16416±9530 |

| | | | | | |
|---|---|---|---|---|---|
| eCF599 | 1 | 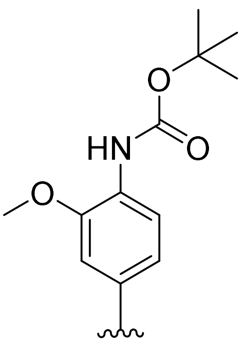 | 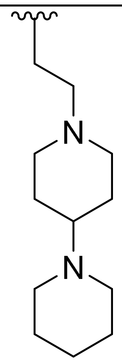 | >30000 | 23005±13436 |
| eCF600 | 1 | 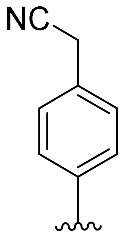 | 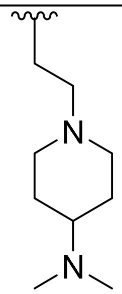 | >30000 | >30000 |
| eCF603 | 1 | 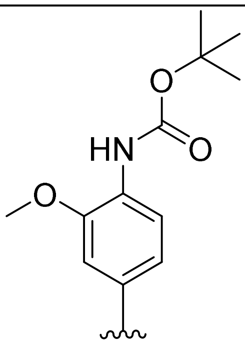 | 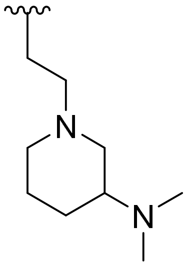 | >30000 | >30000 |
| eCF604 | 1 | 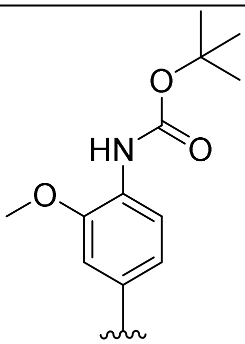 | 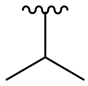 | >30000 | 1034±608 |
| eCF608 | 1 | 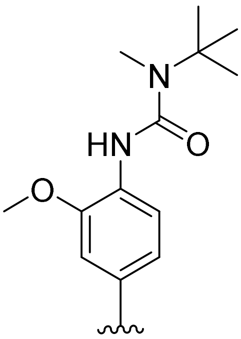 | 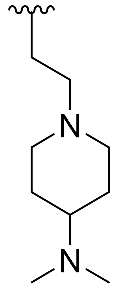 | 21079±3025 | 14402±7529 |

| | | | | | |
|---|---|---|---|---|---|
| eSM104 | 2 | 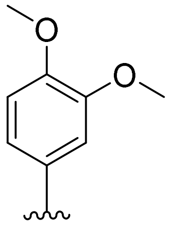 | 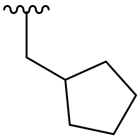 | >30000 | 17452±2141 |
| eSM105 | 2 | 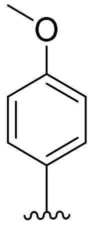 | 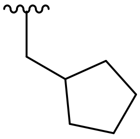 | 20884±1731 | 6359±563 |
| eSM106 | 2 | 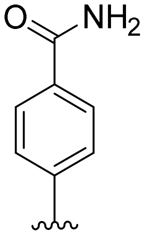 | 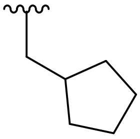 | >30000 | >30000 |
| eSM107 | 2 | 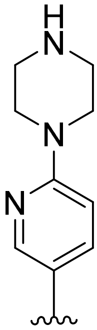 | 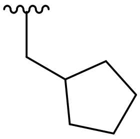 | 3327±166 | 2421±504 |
| eSM108 | 2 | 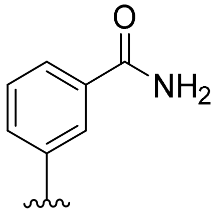 | 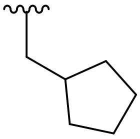 | 23793±2776 | >30000 |
| eSM110 | 2 | 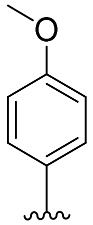 | 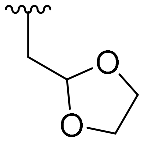 | >30000 | >30000 |
| eSM111 | 2 | 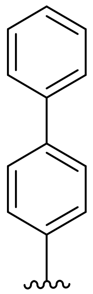 | 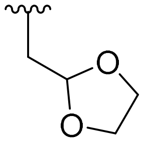 | >30000 | >30000 |

| | | | | | |
|---|---|---|---|---|---|
| eSM112 | 2 | 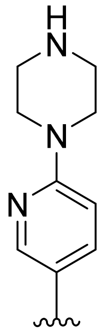 | 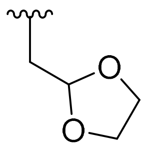 | 21283±2346 | 9260±465 |
| eSM113 | 2 | 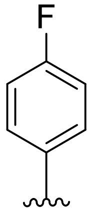 | 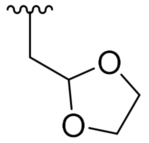 | >30000 | >30000 |
| eSM114 | 2 | 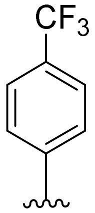 | 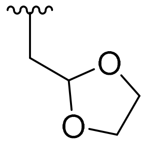 | >30000 | >30000 |
| eSM119 | 2 | 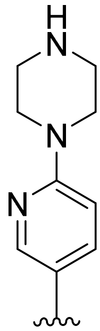 | 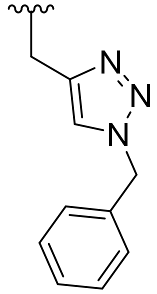 | 9729±1216 | 11273±882 |
| eSM121 | 2 | 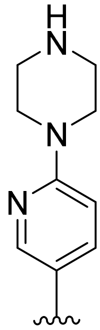 | 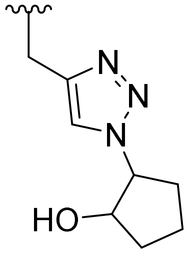 | 16452±1880 | 26344±1815 |
| eSM123 | 2 | 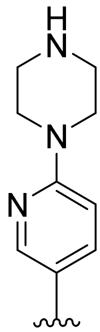 | 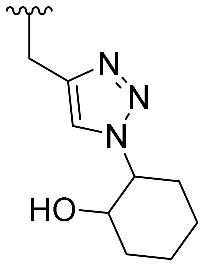 | 25366±4657 | 24201±3600 |

| | | | | | |
|---|---|---|---|---|---|
| eSM127 | 2 | 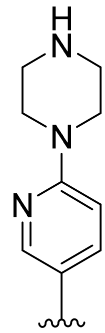 | 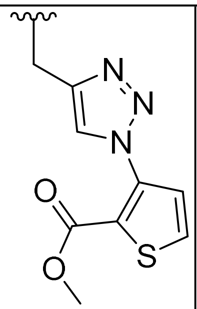 | 9396±2517 | 8195±2310 |
| eSM133 | 2b (NHEt) | 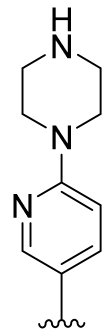 | 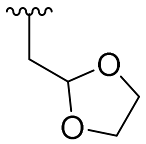 | >30000 | 15618±2745 |
| eSM134 | 2 | 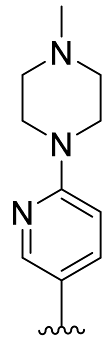 | 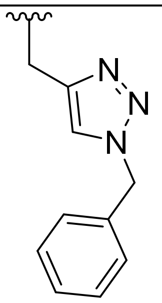 | 18863±5271 | 18830±3569 |
| eSM135 | 2 | 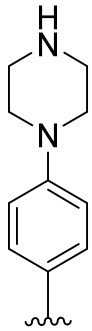 | 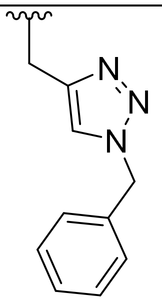 | 13008±291 | 6264±1391 |
| eSM139 | 2 | 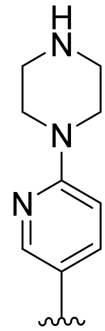 | 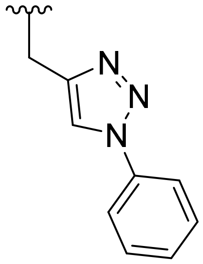 | 6093±1391 | 8603±3387 |

| | | | | | |
|---|---|---|---|---|---|
| eSM143 | 2 | 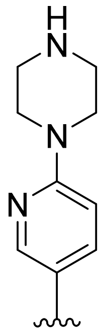 | 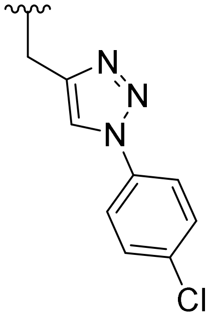 | 3062±833 | 3022±568 |
| eSM145 | 2 | 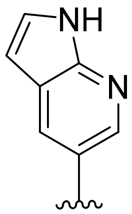 | 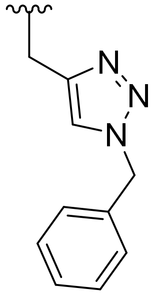 | 14224±557 | 9111±1672 |
| eSM146 | 2 | 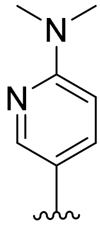 | 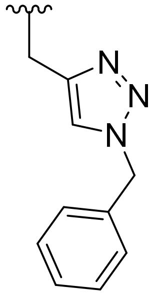 | >30000 | >30000 |
| eSM149 | 2 | 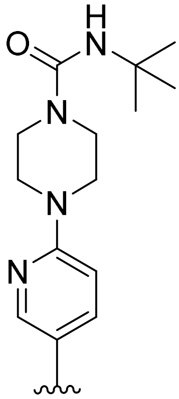 | 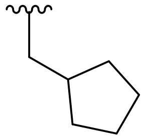 | >30000 | >30000 |
| eSM150 | 2 | 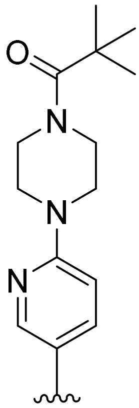 | 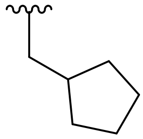 | 22682±9531 | 10124±2998 |

| | | | | | |
|---|---|---|---|---|---|
| eSM153 | 2 | 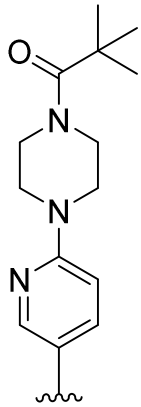 | 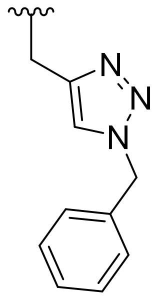 | >30000 | >30000 |
| eSM155 | 2 | 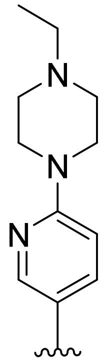 | 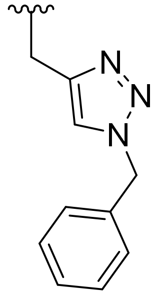 | >30000 | >30000 |
| eSM156 | 2 | 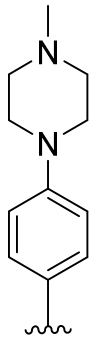 | 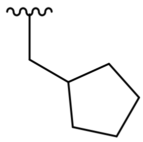 | 7998±3324 | 1774±865 |
| eSM157 | 2 | 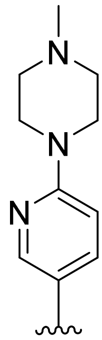 | 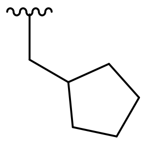 | 7281±2704 | 1707±512 |
| eSM204 | 1 | 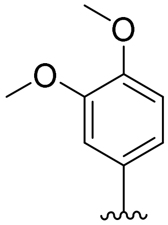 | 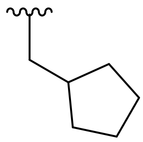 | 8564±564 | 6584±1466 |

| | | | | | | |
|---|---|---|---|---|---|---|
| eSM205 | 1 | 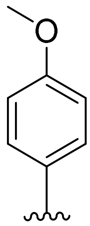 | 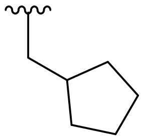 | 14289±6138 | 11940±6496 |
| eSM207 | 1 | 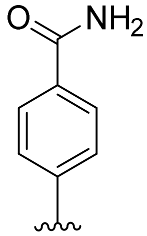 | 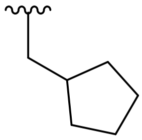 | 19941±6034 | 21273±3278 |
| eSM209 | 1 | 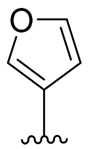 | 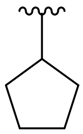 | 23572±2513 | 15607±1449 |
| eSM210 | 1 | 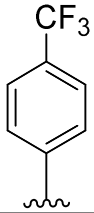 | 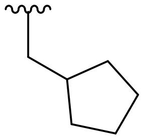 | 24972±3154 | 18829±2693 |
| eSM211 | 1 | 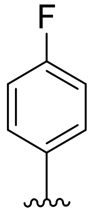 | 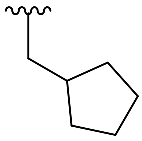 | >30000 | 24602±3253 |
| eSM212 | 1 | 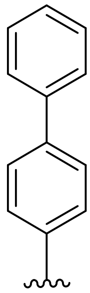 | 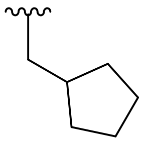 | >30000 | 22793±3126 |

## 1.1. Structures of positive controls TMZ, INK128 and SN-38

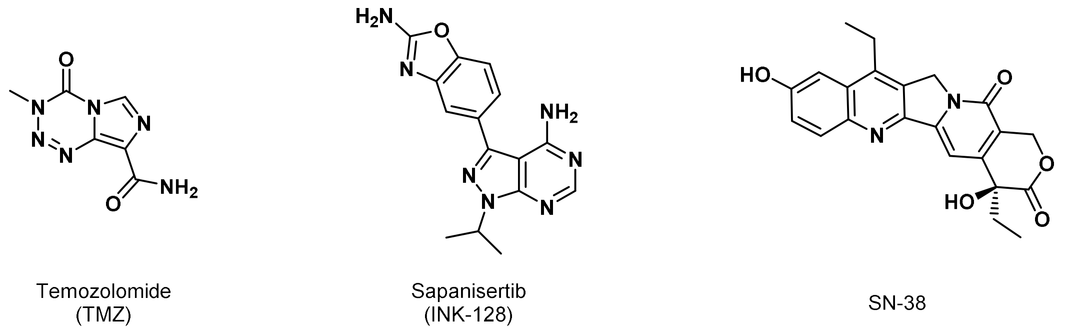


## 2. IC50 values of most potent hits against a panel of recombinant kinases

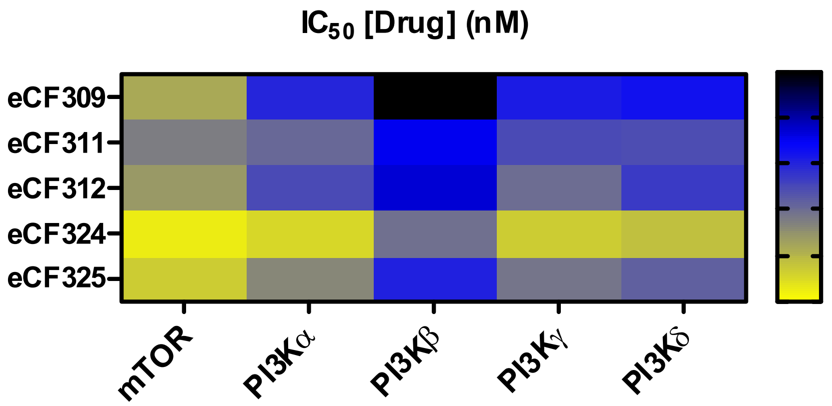


**Figure S1:** Heatmap representation of previously published $IC_{50}$ values of most potent hits against a panel of recombinant kinases.[12]

## 3. Synergy growth inhibition and ZIP synergy scores

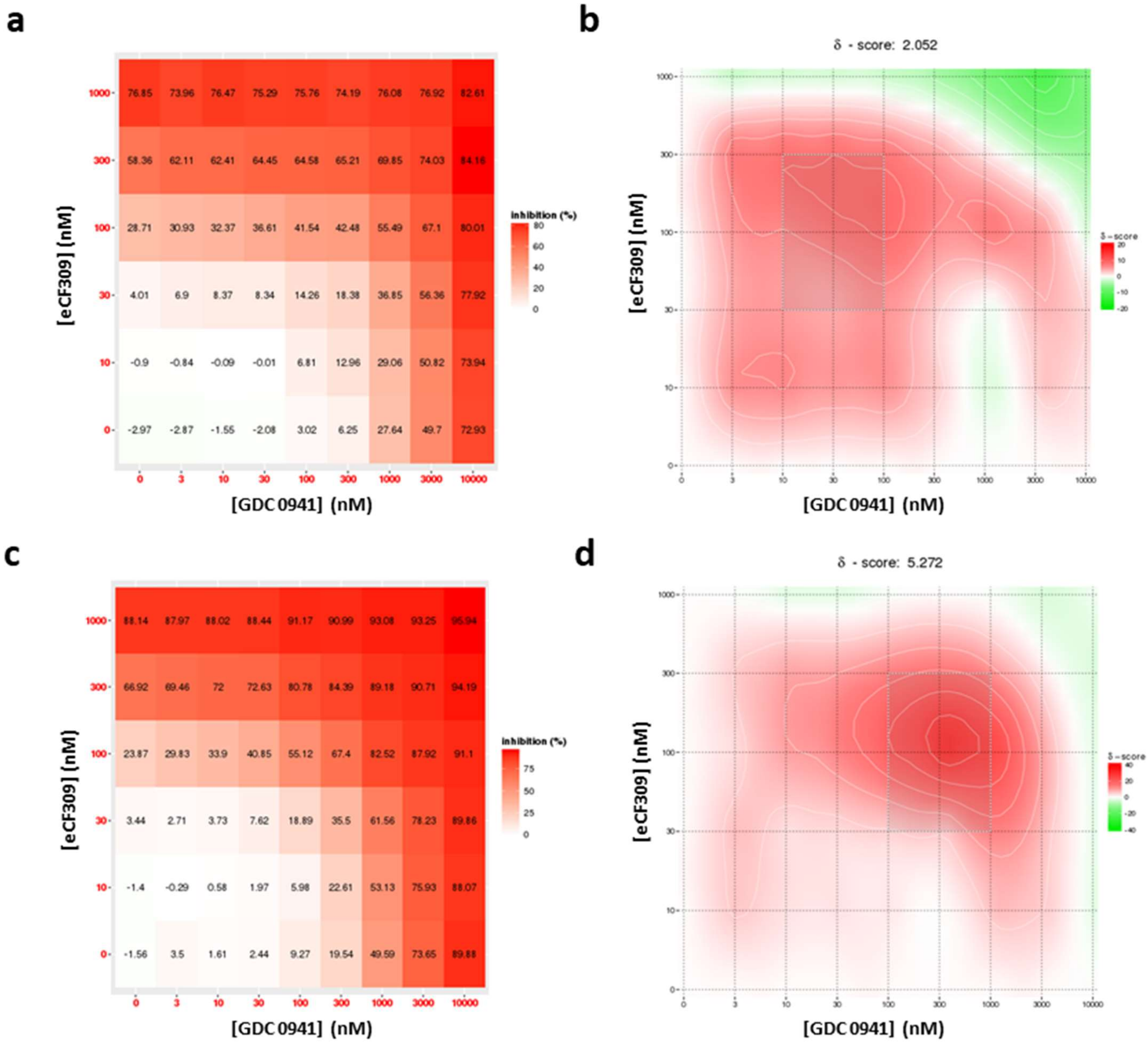


**Figure S2:** Synergy of combinations of the selective mTOR inhibitor **eCF309** and the selective PI3K inhibitor **GDC0941** (GDC) in U87 (**a**, **b**) and T98 cells (**c**, **d**). Cells were treated with combinations of 1000, 300, 100, 30, 10, 0 nM of eCF309 and/or 10000, 3000, 1000, 300, 100, 30, 10, 3, 0 nM of **GDC0941** during 5 d and PrestoBlue reagent was used to detect viability. Combination effect is expressed as inhibition percentage (**a**, **c**) and ZIP[δ] synergy scores (**b**, **d**). Data are means of three independent experiments.

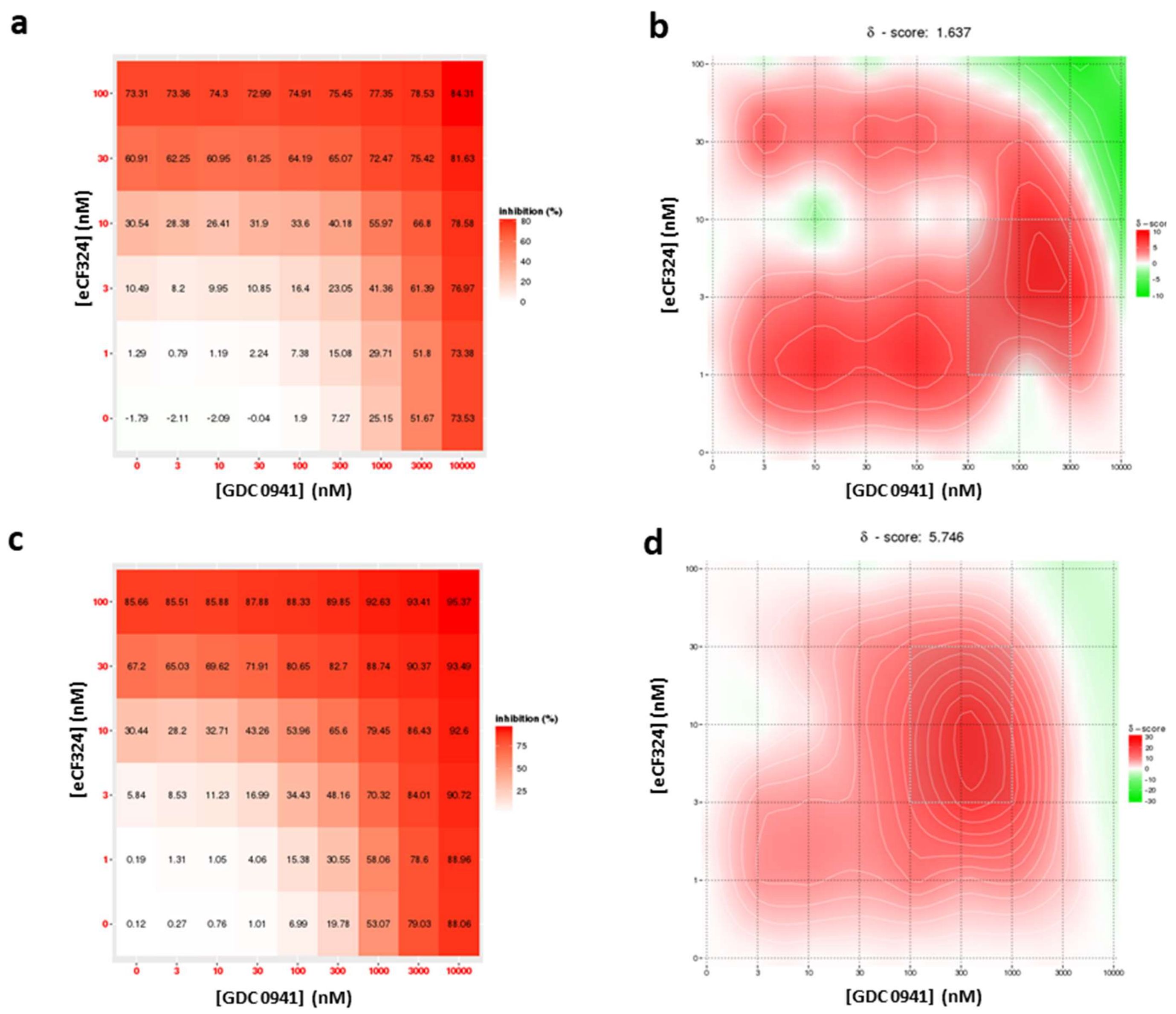


**Figure S3:** Synergy of combinations of the dual mTOR/PI3K inhibitor **eCF324** and the selective PI3K inhibitor **GDC0941** in U87 (**a**, **b**) and T98 cells (**c**, **d**). Cells were treated with combinations of 1000, 300, 100, 30, 10, 0 nM of eCF309 and/or 10000, 3000, 1000, 300, 100, 30, 10, 3, 0 nM of **GDC0941** during 5 d and PrestoBlue reagent was used to detect viability. Combination effect is expressed as inhibition percentage (**a**, **c**) and ZIP(δ) synergy scores (**b**, **d**). Data are means of three independent experiments.

## 4. $GI_{50}$ values of most potent inhibitors on patient-derived cells

**Table S2**. $GI_{50}$ values (nM) calculated for **eCF309**, **eCF311**, **eCF312**, **eCF324**, **eCF325** and controls **INK128**, **GDC0941** and **SN-38** on G317 patient-derived glioma cells. Cells were treated with 10, 3, 1 µM, 300, 100, 30, 10, 3, 1, 0 nM of each inhibitor during 5 d. Cell growth (PrestoBlue) was related to t=0 (before drug treatment), and $GI_{50}$ values were extrapolated from their corresponding sigmoidal dose-response curve (below).

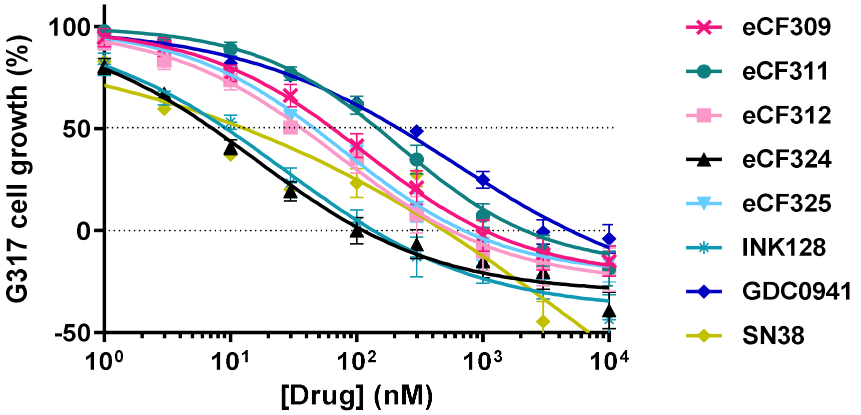


| Cell line | eCF309 | eCF311 | eCF312 | eCF324 | eCF325 | INK128 | GDC0941 | SN38 |
|---|---|---|---|---|---|---|---|---|
| **G317** | 64.9 | 147.1 | 36.9 | 7.2 | 46.4 | 9.3 | 216.0 | 12.9 |

## 5. 3D cell proliferation assay

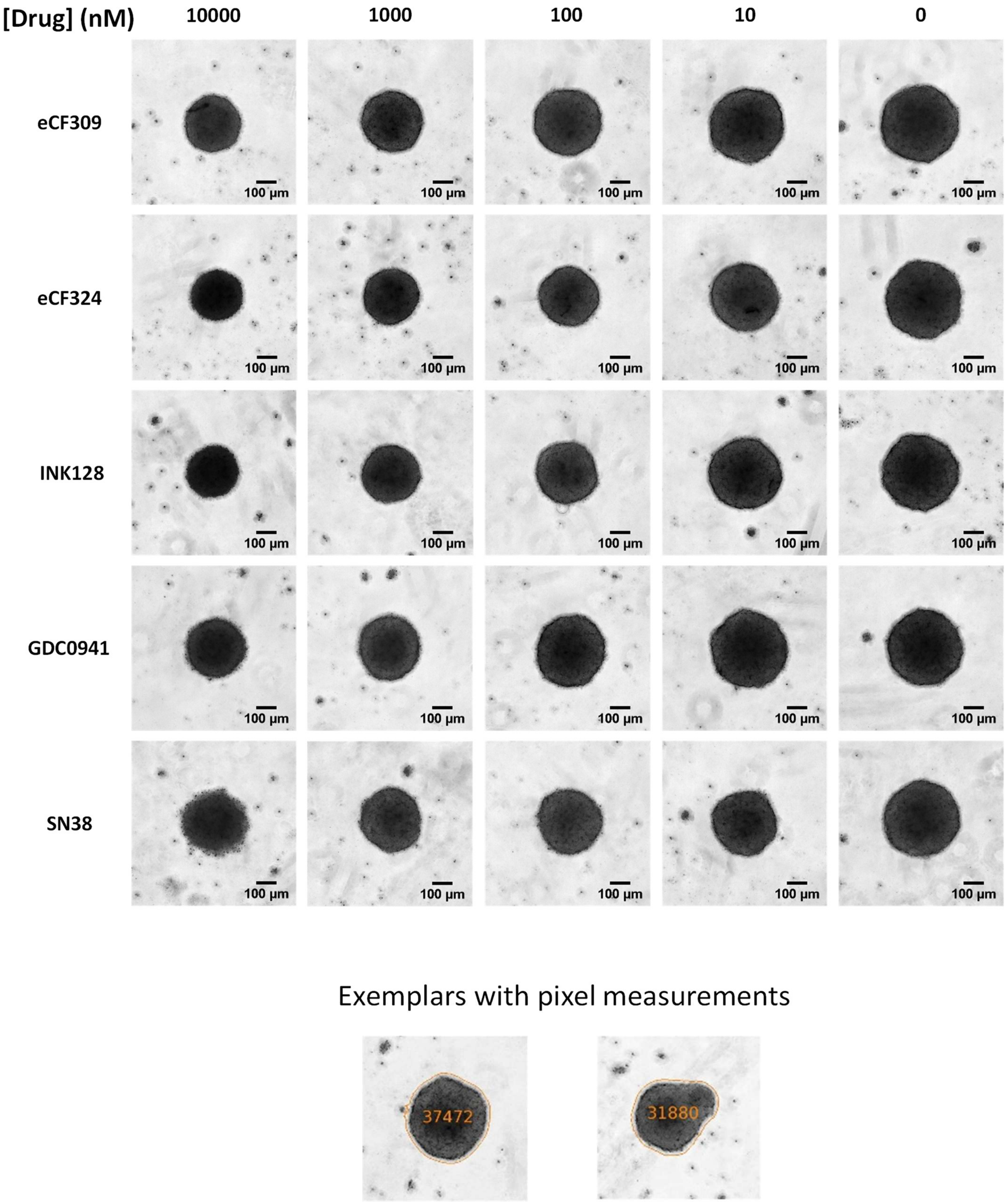


**Figure S4.** (a) Representative images of G317 spheroids on day 10. Spheroids were treated with 10000, 1000, 100, 10, 0 nM of each inhibitor during 8 days, images were acquired using ImageXpress (Scale bar 100µm).

## 6. $GI_{50}$ values of most potent inhibitors on glioma vs. noncancerous brain cells

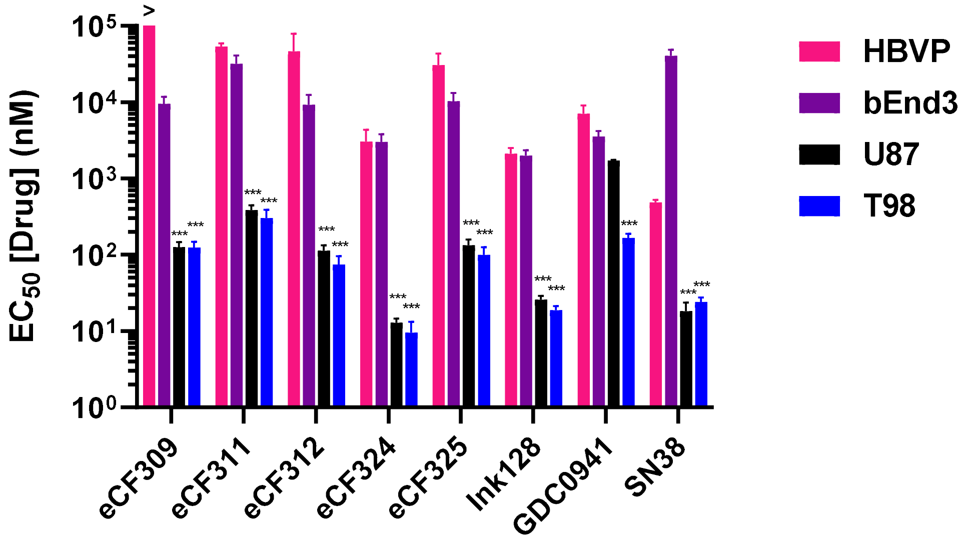


**Figure S5.** Comparison of $EC_{50}$ values of the hits and positive controls on glioma U87 and T98 cell lines vs. non-cancerous human brain vascular pericytes (HBVP) and murine brain endothelial cells (bEnd3). Cells were treated with each inhibitor at concentrations ranging from 1 to 30,000 nM and PrestoBlue assay used at day 5 to detect viability. Data are means ± SEM of 3 independent experiments in triplicates. ***$P < 0.001$ compared to both non-cancerous cell lines (two-way ANOVA, Tukey post hoc test).

## 7. General chemistry methods

Syntheses were carried out in closed reaction vessels in a Biotage® Initiator Third Generation microwave reactor. Commercially available chemicals and anhydrous solvents that were purchased from a range of suppliers, including: Acros Organics, Alfa Aesar, Fisher Scientific, Fluorochem, Matrix Scientific, Sigma Aldrich and VWR International. Reactions were monitored by thin-layer chromatography (TLC) using Merck TLC Silica gel 60 F254 plates, cut to be approximately 5 cm x 10 cm. Detection of the spots was obtained through the use of a potassium permanganate dip and drying using a heat gun, or by visualisation using UV light at 254 nm. Compounds were purified by flash column chromatography using 220-240 mesh silica gel, purchased from Sigma Aldrich, and commercially available solvents, carried out in a glass column fitted with frit and PET tap.

NMR spectra were recorded at ambient temperature in a 500 MHz Bruker Avance III spectrometer, at the University of Edinburgh's School of Chemistry. Samples were dissolved in deuterated solvents commercially available from Sigma-Aldrich. $^1$H-NMR spectra: chemical shifts are reported in parts per million (ppm) relative to the solvent peak. The data is presented as follows: chemical shift, integration, multiplicity (s = singlet, d = doublet, t = triplet, q = quartet, m = multiplet), coupling constants as a J value in Hertz (Hz) and interpretation. The number of protons (n) for a given resonance is indicated as nH and is based on spectral integration values. The data is presented as follows: chemical shift and assignment. High resolution mass spectra were recorded by the MS Department of the University of Edinburgh on a Thermo MAT 900 XLP high resolution, double focusing mass spectrometer.

## 8. Structures of new derivatives synthesized in this work

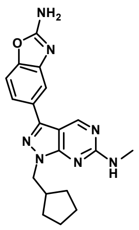

**5-(1-(cyclopentylmethyl)-6-(methylamino)-1*H*-pyrazolo[3,4-*d*]pyrimidin-3-yl)benzo[*d*]oxazol-2-amine**
Chemical Formula: $C_{19}H_{21}N_7O$
Molecular Weight: 363.43

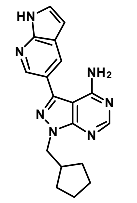

**1-(cyclopentylmethyl)-3-(1*H*-pyrrolo[2,3-*b*]pyridin-5-yl)-1*H*-pyrazolo[3,4-*d*]pyrimidin-4-amine**
Chemical Formula: $C_{18}H_{19}N_7$
Molecular Weight: 333.40

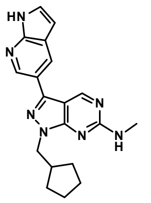

**1-(cyclopentylmethyl)-*N*-methyl-3-(1*H*-pyrrolo[2,3-*b*]pyridin-5-yl)-1*H*-pyrazolo[3,4-*d*]pyrimidin-6-amine**
Chemical Formula: $C_{19}H_{21}N_7$
Molecular Weight: 347.43

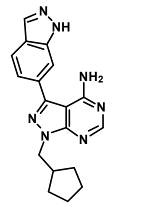

**1-(cyclopentylmethyl)-3-(1*H*-indazol-6-yl)-1*H*-pyrazolo[3,4-*d*]pyrimidin-4-amine**
Chemical Formula: $C_{18}H_{19}N_7$
Molecular Weight: 333.40

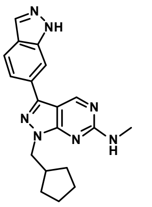

**1-(cyclopentylmethyl)-3-(1*H*-indazol-6-yl)-*N*-methyl-1*H*-pyrazolo[3,4-*d*]pyrimidin-6-amine**
Chemical Formula: $C_{19}H_{21}N_7$
Molecular Weight: 347.43

## 9. Synthesis of new derivatives

### eDB001
### 5-(1-(cyclopentylmethyl)-6-(methylamino)-1*H*-pyrazolo[3,4-*d*]pyrimidin-3-yl)benzo[*d*]oxazol-2-amine

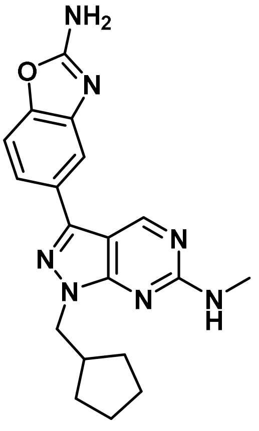

1-(cyclopentylmethyl)-3-iodo-*N*-methyl-1*H*-pyrazolo[3,4-*d*]pyrimidin-6-amine (77.1 mg, 0.216 mmol) was added to a 5 mL microwave vial equipped with a stirrer bar. To the vial were added, 6-(4,4,5,5-tetramethyl-1,3,2-dioxoborolan-2-yl)benzo[*d*]oxazol-2-amine (78.5 mg, 0.302 mmol, 1.4 eq), palladium (II) acetate (4.8 mg, 0.021 mmol, 10 mol%), triphenylphosphine (12.6 mg, 0.048 mmol, 22 mol%) and potassium carbonate (46.4 mg, 0.336 mmol, 1.6 eq). The reagents were suspended in 9:1 dioxane:water (5 mL). The vial was sealed with a septum cap and placed into a microwave reactor. The reaction was heated to and stirred at 120 °C for 1 h under microwave irradiation, yielding a golden-brown mixture.

The reaction was poured into water (30 mL) and partitioned with EtOAc (50 mL). The organic layer was collected, and the aqueous layer was washed with EtOAc (3 x 50 mL). The organic layers were combined and washed with water (40 mL) and brine (50 mL). The fraction was dried over $MgSO_4$ and concentrated *in vacuo* to give the crude. The crude was purified by flash column chromatography on silica using a 0-10 % MeOH/DCM eluent gradient. The appropriate fractions by TLC were combined and concentrated to give the product, 5-(1-(cyclopentylmethyl)-6-(methylamino)-1*H*-pyrazolo[3,4-*d*]pyrimidin-3-yl)benzo[*d*]oxazol-2-amine (38.1 mg, 0.104 mmol, 49 %) as a white solid.

$^{1}$H NMR (500 MHz, DMSO-$d_6$) δ 9.08 (br. s., 1H), 7.72 (d, $J$ = 1.58 Hz, 1H), 7.59 (dd, $J$ = 1.73, 8.28 Hz, 1H), 7.49 (s, 2H), 7.41 (d, $J$ = 8.20 Hz, 2H), 4.17 (d, $J$ = 7.01 Hz, 2H), 2.88 (d, $J$ = 4.49 Hz, 3H), 2.52 - 2.58 (m, 1H), 1.63 (br. s., 4H), 1.45 - 1.56 (m, 2H), 1.30 - 1.41 (m, 2H)

$^{13}$C NMR (126 MHz, DMSO-$d_6$) δ 163.3, 161.2, 155.7, 153.8, 143.1, 119.0, 113.1, 108.7, 58.9, 49.9, 29.7, 24.5

MS: [M+H] = 364.19

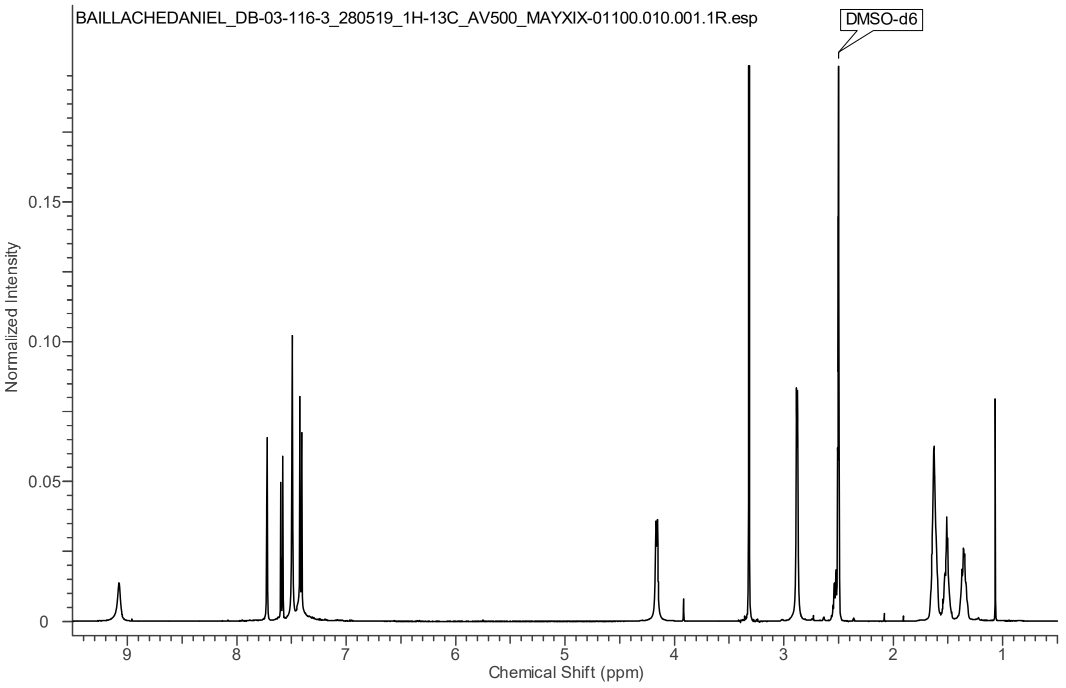


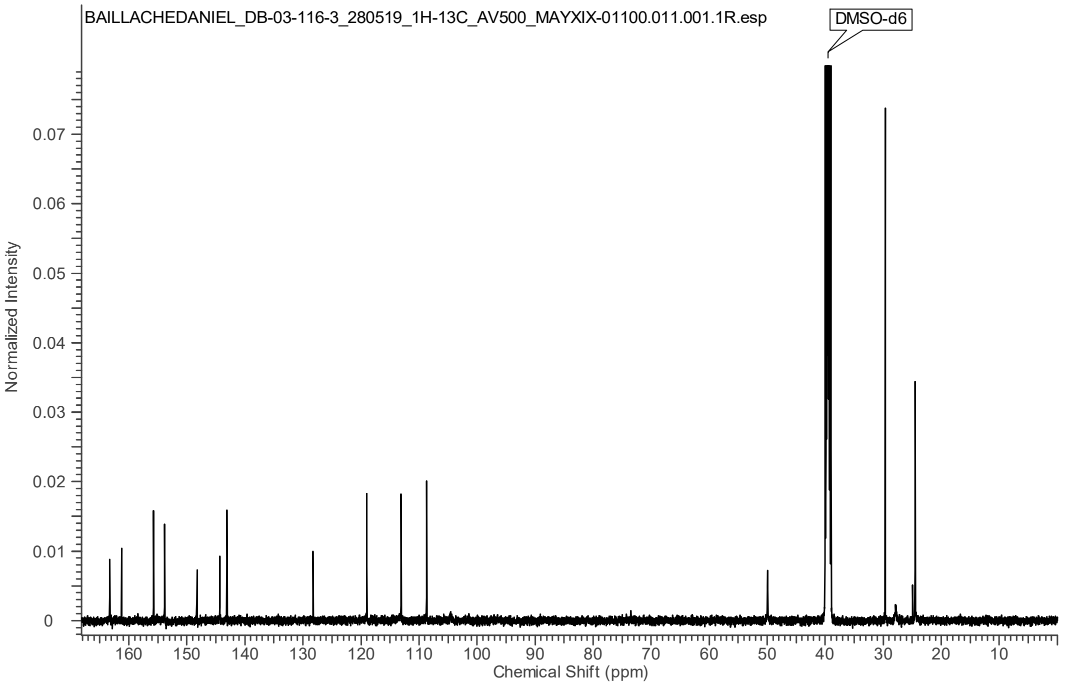

**eDB002**

**1-(cyclopentylmethyl)-3-(1*H*-pyrrolo[2,3-*b*]pyridin-5-yl)-1*H*-pyrazolo[3,4-*d*]pyrimidin-4-amine**

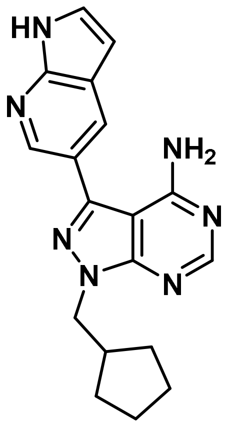

1-(cyclopentylmethyl)-3-iodo-1*H*-pyrazolo[3,4-*d*]pyrimidin-4-amine (108.7 mg, 0.316 mmol) was added to a 5 mL microwave vial equipped with a stirrer bar. To the vial were added, 7-azaindole-5-boronic acid pinacol ester (115.0 mg, 0.471 mmol, 1.5 eq), palladium (II) acetate (4.0 mg, 0.018 mmol, 6 mol%), triphenylphosphine (17.1 mg, 0.065 mmol, 20 mol%) and potassium carbonate (68.2 mg, 0.493 mmol, 1.6 eq). The reagents were suspended in 9:1 dioxane:water (5 mL). The vial was sealed with a septum cap and placed into a microwave reactor. The reaction was heated to and stirred at 120 °C for 1 h under microwave irradiation, yielding a brown mixture.

The reaction was poured into water (20 mL) and partitioned with EtOAc (40 mL). The organic layer was collected, and the aqueous layer was washed with EtOAc (3 x 40 mL). The organic layers were combined and washed with water (20 mL) and brine (50 mL). The fraction was dried over $MgSO_4$ and concentrated *in vacuo* to give the crude. The crude was purified by flash column chromatography on silica using a 0-15 % MeOH/DCM eluent gradient. The appropriate fractions by TLC were combined and concentrated to give the product, 1-(cyclopentylmethyl)-3-(1H-pyrrolo[2,3-b]pyridin-5-yl)-1H-pyrazolo[3,4-d]pyrimidin-4-amine (85.7 mg, 0.257 mmol, 81 %) as a pink fluffy solid.

$^1$H NMR (400 MHz, DMSO-$d_6$) δ 11.82 (br. s., 1H), 8.47 (d, *J* = 2.08 Hz, 1H), 8.25 (s, 1H), 8.19 (d, *J* = 1.58 Hz, 1H), 7.56 (dd, *J* = 2.68, 3.25 Hz, 1H), 6.64 - 7.10 (m, 2H), 6.56 (dd, *J* = 1.86, 3.44 Hz, 1H), 4.28 (d, *J* = 7.38 Hz, 2H), 2.53 (quin, *J* = 7.10 Hz, 1H), 1.57 - 1.68 (m, 4H), 1.44 - 1.56 (m, 2H), 1.29 - 1.41 (m, 2H)

$^{13}$C NMR (126 MHz, DMSO-$d_6$) δ 158.3, 155.7, 154.3, 148.4, 142.3, 142.3, 127.8, 127.1, 120.9, 119.5, 100.4, 97.4, 50.7, 30.7, 29.6, 24.6

MS: [M+H] = 334.18

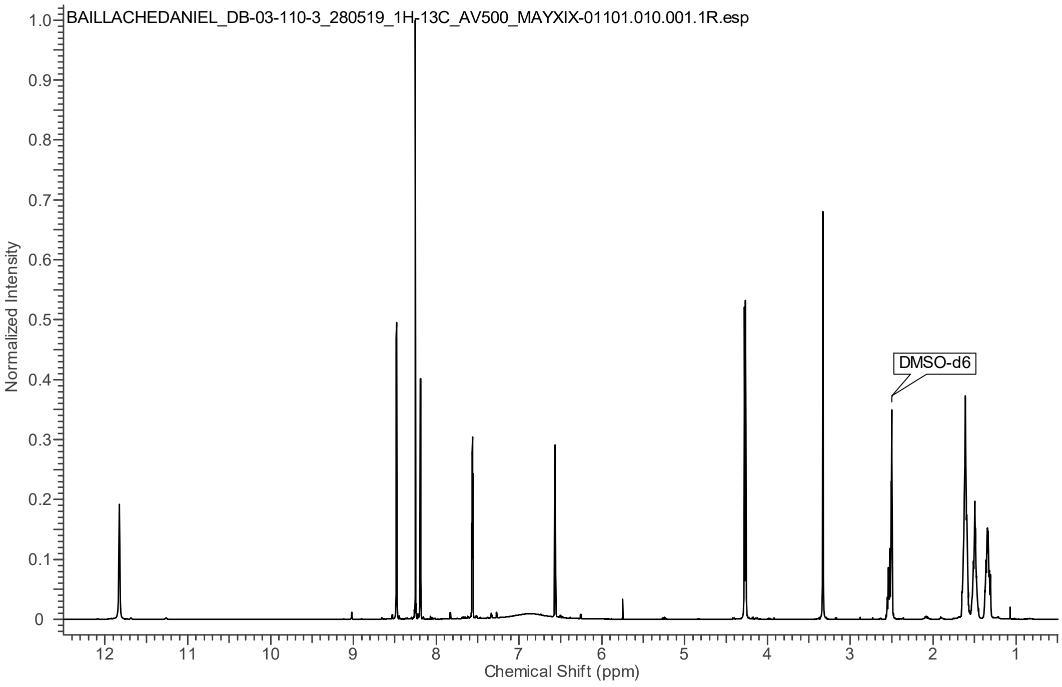
BAILLACHEDANIEL_DB-03-110-3_280519_1H-13C_AV500_MAYXIX-01101.010.001.1R.esp
DMSO-d6
Normalized Intensity
Chemical Shift (ppm)

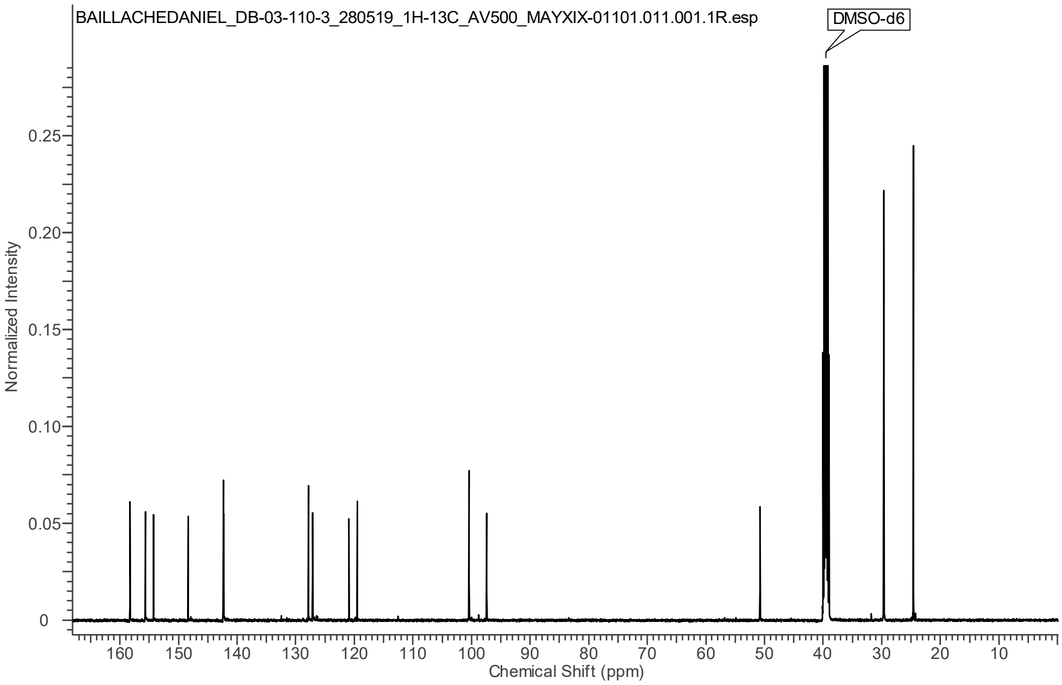
BAILLACHEDANIEL_DB-03-110-3_280519_1H-13C_AV500_MAYXIX-01101.011.001.1R.esp
DMSO-d6
Normalized Intensity
Chemical Shift (ppm)

**eDB003**
**1-(cyclopentylmethyl)-*N*-methyl-3-(1*H*-pyrrolo[2,3-*b*]pyridin-5-yl)-1*H*-pyrazolo[3,4-*d*]pyrimidin-6-amine**

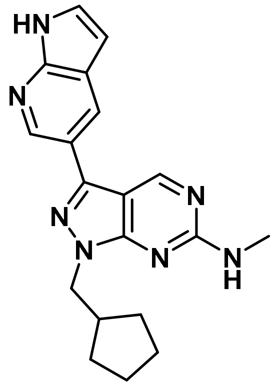

1-(cyclopentylmethyl)-3-iodo-*N*-methyl-1*H*-pyrazolo[3,4-*d*]pyrimidin-6-amine (95.2 mg, 0.267 mmol) was added to a 5 mL microwave vial equipped with a stirrer bar. To the vial were added, 7-azaindole-5-boronic acid pinacol ester (99.8 mg, 0.409 mmol, 1.5 eq), palladium (II) acetate (4.0 mg, 0.018 mmol, 7 mol%), triphenylphosphine (22.9 mg, 0.087 mmol, 32 mol%) and potassium carbonate (69.7 mg, 0.504 mmol, 1.8 eq). The reagents were suspended in 9:1 dioxane:water (5 mL). The vial was sealed with a septum cap and placed into a microwave reactor. The reaction was heated to and stirred at 120 °C for 1 h under microwave irradiation, yielding a dark brown mixture.

The reaction was poured into water (20 mL) and partitioned with EtOAc (40 mL). The organic layer was collected, and the aqueous layer was washed with EtOAc (3 x 40 mL). The organic layers were combined and washed with water (2 x 20 mL) and brine (50 mL). The fraction was dried over $MgSO_4$ and concentrated *in vacuo* to give the crude. The crude was purified by flash column chromatography on silica using a 0-15 % MeOH/DCM eluent gradient. The appropriate fractions by TLC were combined and concentrated to give the product, 1-(cyclopentylmethyl)-*N*-methyl-3-(1*H*-pyrrolo[2,3-*b*]pyridin-5-yl)-1*H*-pyrazolo[3,4-*d*]pyrimidin-6-amine (82.1 mg, 0.236 mmol, 89 %) as a light brown solid.

$^{1}$H NMR (400 MHz, DMSO-$d_6$) δ 11.78 (br. s., 1H), 9.16 (br. s., 1H), 8.82 (d, *J* = 2.08 Hz, 1H), 8.51 (d, *J* = 2.02 Hz, 1H), 7.53 (dd, *J* = 2.65, 3.22 Hz, 1H), 7.45 (br. s., 1H), 6.55 (dd, *J* = 1.86, 3.44 Hz, 1H), 4.19 (d, *J* = 7.44 Hz, 2H), 2.89 (d, *J* = 4.67 Hz, 3H), 2.55 (quin, *J* = 7.10 Hz, 1H), 1.65 (br. s., 4H), 1.46 - 1.57 (m, 2H), 1.31 - 1.43 (m, 2H)

$^{13}$C NMR (126 MHz, DMSO-$d_6$) δ 155.7, 154.0, 148.4, 141.9, 141.1, 127.1, 126.0, 120.6, 119.6, 100.5, 50.0, 29.7, 24.5

MS: [M+H] = 348.19

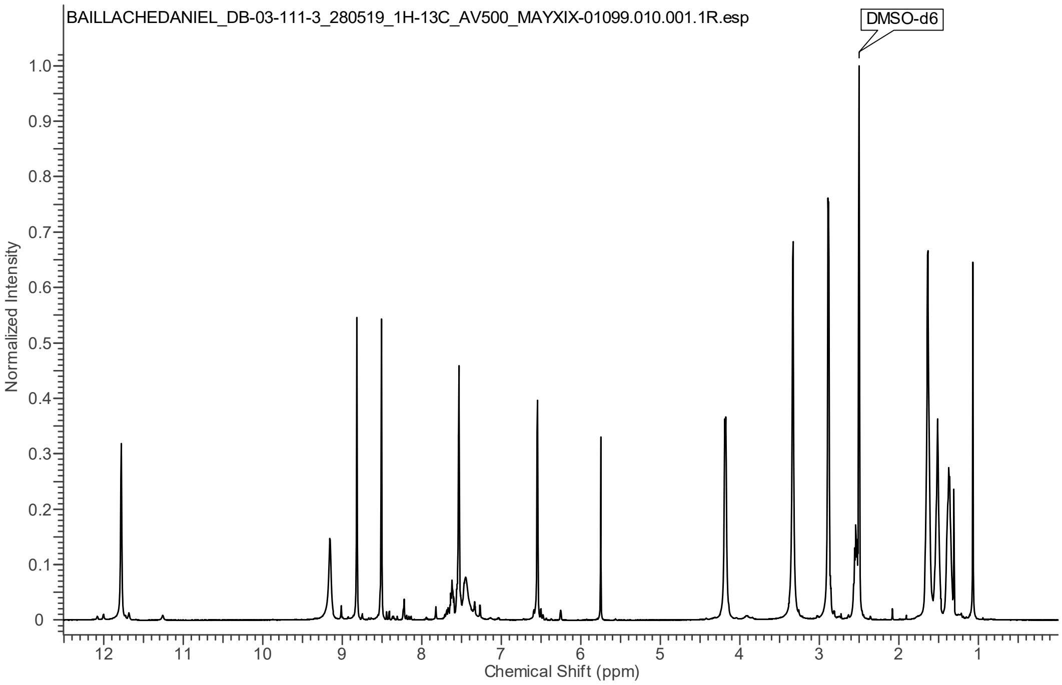
BAILLACHEDANIEL_DB-03-111-3_280519_1H-13C_AV500_MAYXIX-01099.010.001.1R.esp
DMSO-d6
Normalized Intensity
Chemical Shift (ppm)

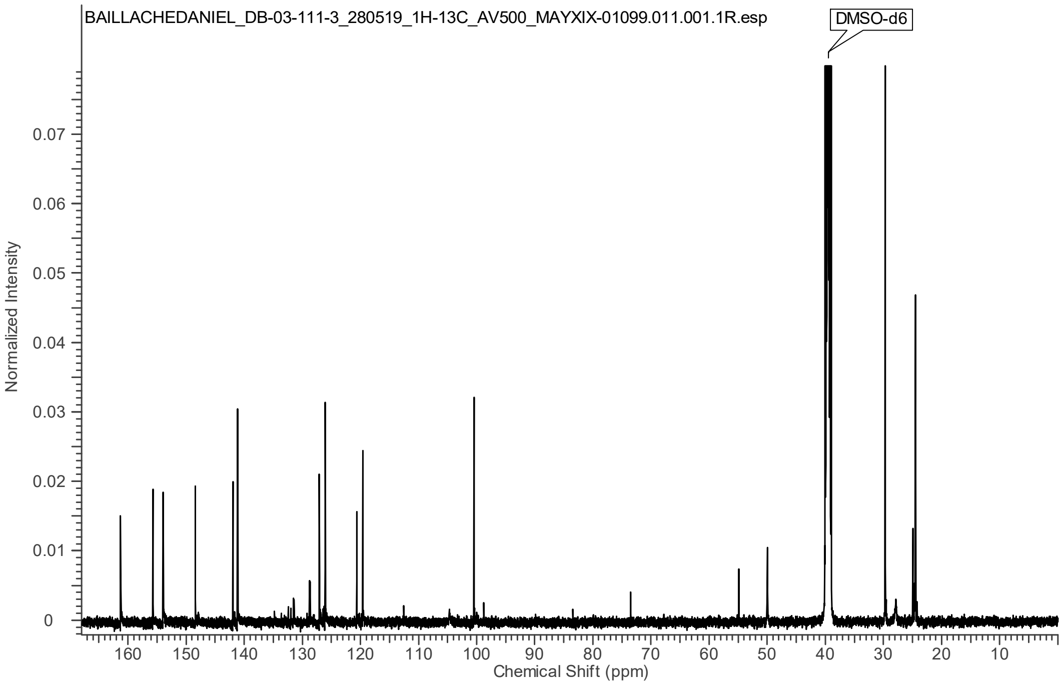
BAILLACHEDANIEL_DB-03-111-3_280519_1H-13C_AV500_MAYXIX-01099.011.001.1R.esp
DMSO-d6
Normalized Intensity
Chemical Shift (ppm)

**eDB004**

**1-(cyclopentylmethyl)-3-(1*H*-indazol-6-yl)-1*H*-pyrazolo[3,4-*d*]pyrimidin-4-amine**

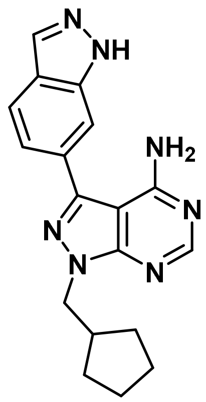


1-(cyclopentylmethyl)-3-iodo-1*H*-pyrazolo[3,4-*d*]pyrimidin-4-amine (130.5 mg, 0.380 mmol) was added to a 5 mL microwave vial equipped with a stirrer bar. To the vial were added, 1*H*-indazole-6-boronic acid (93.7 mg, 0.579 mmol, 1.5 eq), palladium (II) acetate (9.4 mg, 0.042 mmol, 11 mol%), triphenylphosphine (20.2 mg, 0.077 mmol, 20 mol%) and potassium carbonate (80.6 mg, 0.583 mmol, 1.5 eq). The reagents were suspended in 9:1 dioxane:water (5 mL). The vial was sealed with a septum cap and placed into a microwave reactor. The reaction was heated to and stirred at 120 °C for 1 h under microwave irradiation, yielding a golden-brown mixture.

The reaction was poured into water (40 mL) and partitioned with EtOAc (50 mL). The organic layer was collected, and the aqueous layer was washed with EtOAc (4 x 40 mL). The organic layers were combined and washed with water (50 mL) and brine (50 mL). The fraction was dried over $MgSO_4$ and concentrated *in vacuo* to give the crude. The crude was purified by flash column chromatography on silica using a 0-10 % MeOH/DCM eluent gradient. The appropriate fractions by TLC were combined and concentrated to give the product, 1-(cyclopentylmethyl)-3-(1*H*-indazol-6-yl)-1*H*-pyrazolo[3,4-*d*]pyrimidin-4-amine (75.4 mg, 0.226 mmol, 60 %) as a yellow solid.

$^{1}$H NMR (500 MHz, DMSO-$d_6$) δ 13.21 (s, 1H), 8.26 (s, 1H), 8.15 (t, *J* = 1.22 Hz, 1H), 7.91 (d, *J* = 8.28 Hz, 1H), 7.77 (d, *J* = 0.95 Hz, 1H), 7.44 (dd, *J* = 1.34, 8.28 Hz, 1H), 4.28 (d, *J* = 7.41 Hz, 2H), 2.53 (spt, *J* = 7.30 Hz, 1H), 1.57 - 1.67 (m, 4H), 1.45 - 1.56 (m, 2H), 1.30 - 1.40 (m, 2H)

$^{13}$C NMR (126 MHz, DMSO-$d_6$) δ 158.2, 155.6, 154.3, 143.7, 140.1, 133.5, 130.5, 122.7, 121.2, 120.8, 109.9, 97.2, 50.8, 48.6, 29.6, 24.6

MS: [M+H] = 334.18

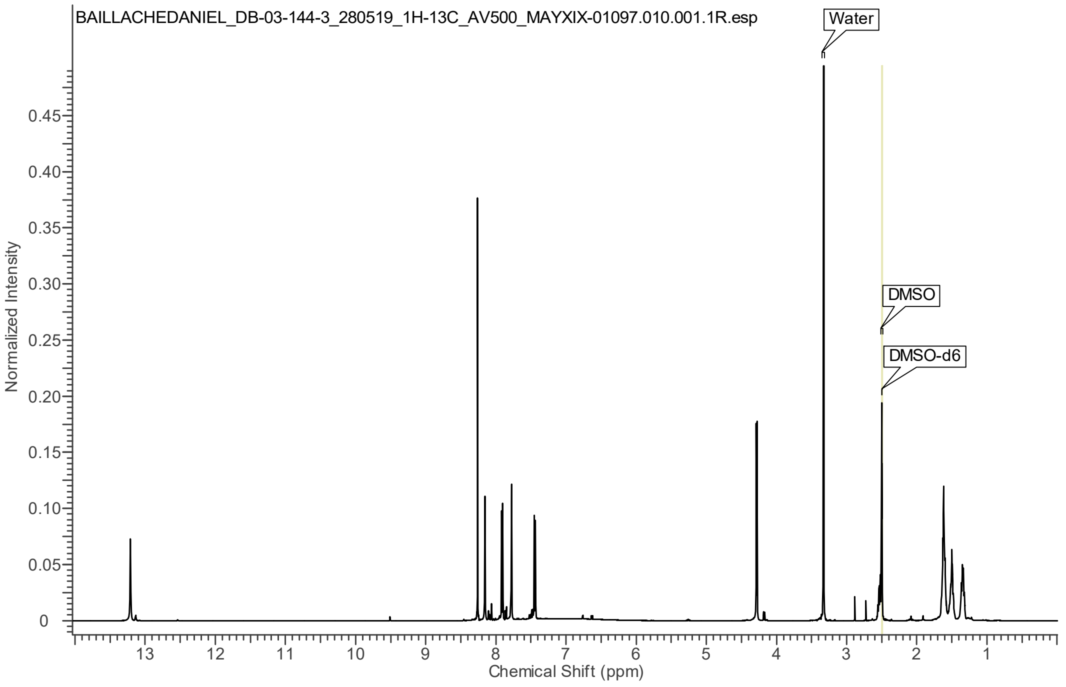
BAILLACHEDANIEL_DB-03-144-3_280519_1H-13C_AV500_MAYXIX-01097.010.001.1R.esp
Water
DMSO
DMSO-d6
Normalized Intensity
Chemical Shift (ppm)

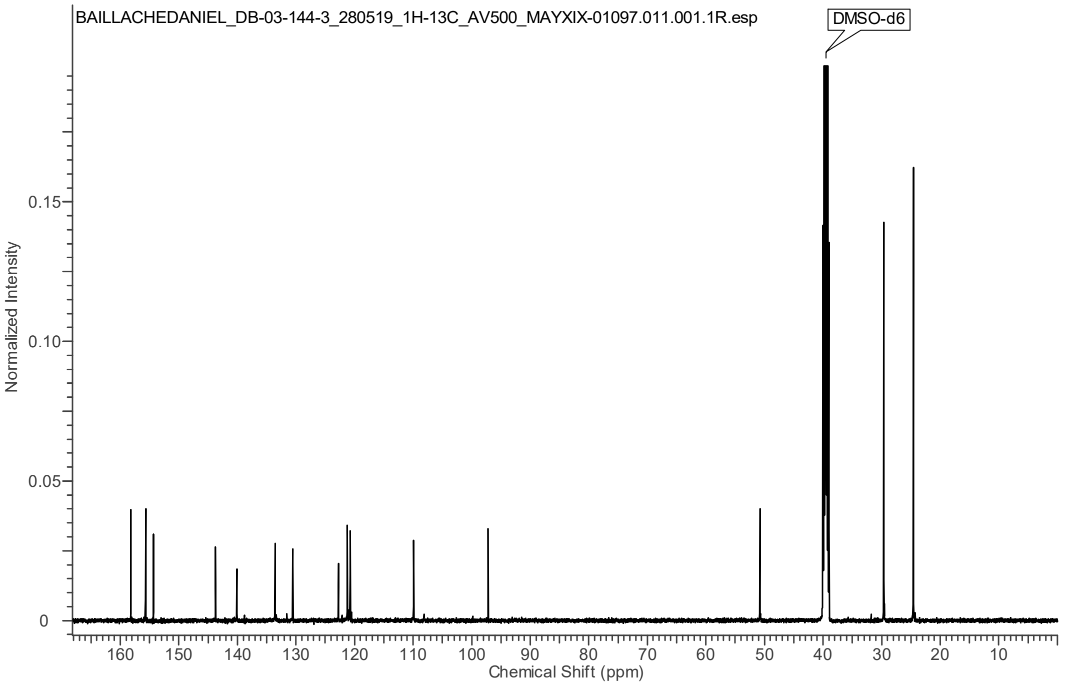
BAILLACHEDANIEL_DB-03-144-3_280519_1H-13C_AV500_MAYXIX-01097.011.001.1R.esp
DMSO-d6
Normalized Intensity
Chemical Shift (ppm)

**eDB005**

**1-(cyclopentylmethyl)-3-(1*H*-indazol-6-yl)-*N*-methyl-1*H*-pyrazolo[3,4-*d*]pyrimidin-6-amine**

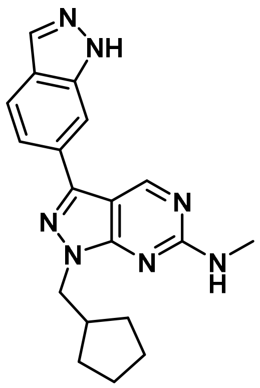


1-(cyclopentylmethyl)-3-iodo-*N*-methyl-1*H*-pyrazolo[3,4-*d*]pyrimidin-6-amine (113.5 mg, 0.318 mmol) was added to a 5 mL microwave vial equipped with a stirrer bar. To the vial were added, 1*H*-indazole-6-boronic acid (77.9 mg, 0.481 mmol, 1.5 eq), palladium (II) acetate (4.1 mg, 0.018 mmol, 6 mol%), triphenylphosphine (19.2 mg, 0.073 mmol, 23 mol%) and potassium carbonate (66.4 mg, 0.480 mmol, 1.5 eq). The reagents were suspended in 9:1 dioxane:water (5 mL). The vial was sealed with a septum cap and placed into a microwave reactor. The reaction was heated to and stirred at 120 °C for 1 h under microwave irradiation, yielding a dark brown mixture.

The reaction was poured into water (40 mL) and partitioned with EtOAc (50 mL). The organic layer was collected, and the aqueous layer was washed with EtOAc (3 x 50 mL). The organic layers were combined and washed with water (50 mL) and brine (50 mL). The fraction was dried over $MgSO_4$ and concentrated *in vacuo* to give the crude. The crude was purified by flash column chromatography on silica using a 0-10 % MeOH/DCM eluent gradient. The appropriate fractions by TLC were combined and concentrated to give the product, 1-(cyclopentylmethyl)-3-(1*H*-indazol-6-yl)-*N*-methyl-1*H*-pyrazolo[3,4-*d*]pyrimidin-6-amine (86.5 mg, 0.246 mmol, 78 %) as a white solid.

$^{1}$H NMR (500 MHz, DMSO-$d_6$) δ 13.11 (s, 1H), 9.14 (br. s., 1H), 8.12 (t, *J* = 1.18 Hz, 1H), 8.07 (d, *J* = 0.87 Hz, 1H), 7.86 - 7.88 (m, 1H), 7.75 (dd, *J* = 1.34, 8.35 Hz, 1H), 7.43 - 7.55 (m, 1H), 4.20 (d, *J* = 6.78 Hz, 2H), 2.89 (d, *J* = 4.41 Hz, 3H), 2.55 (spt, *J* = 7.20 Hz, 1H), 1.58 - 1.69 (m, 4H), 1.47 - 1.57 (m, 2H), 1.31 - 1.44 (m, 2H)

$^{13}$C NMR (126 MHz, DMSO-$d_6$) δ 161.3, 155.9, 153.8, 143.0, 140.1, 133.6, 130.1, 122.8, 121.1, 119.3, 107.7, 50.0, 29.7, 24.5

MS: [M+H] = 348.19

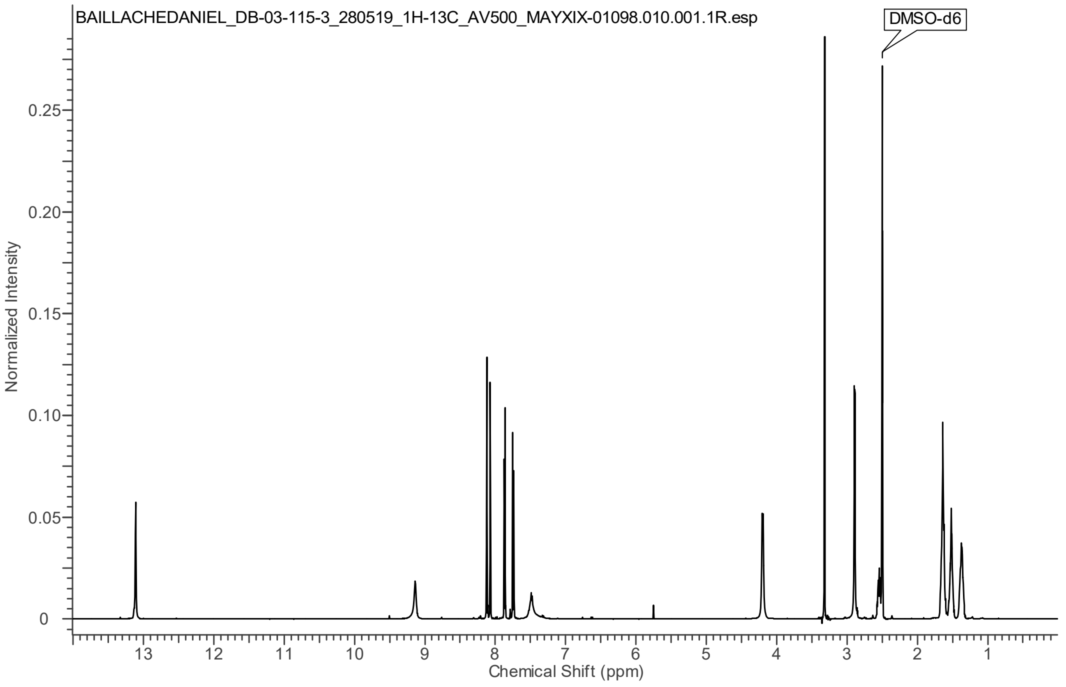
BAILLACHEDANIEL_DB-03-115-3_280519_1H-13C_AV500_MAYXIX-01098.010.001.1R.esp
DMSO-d6
Normalized Intensity
Chemical Shift (ppm)

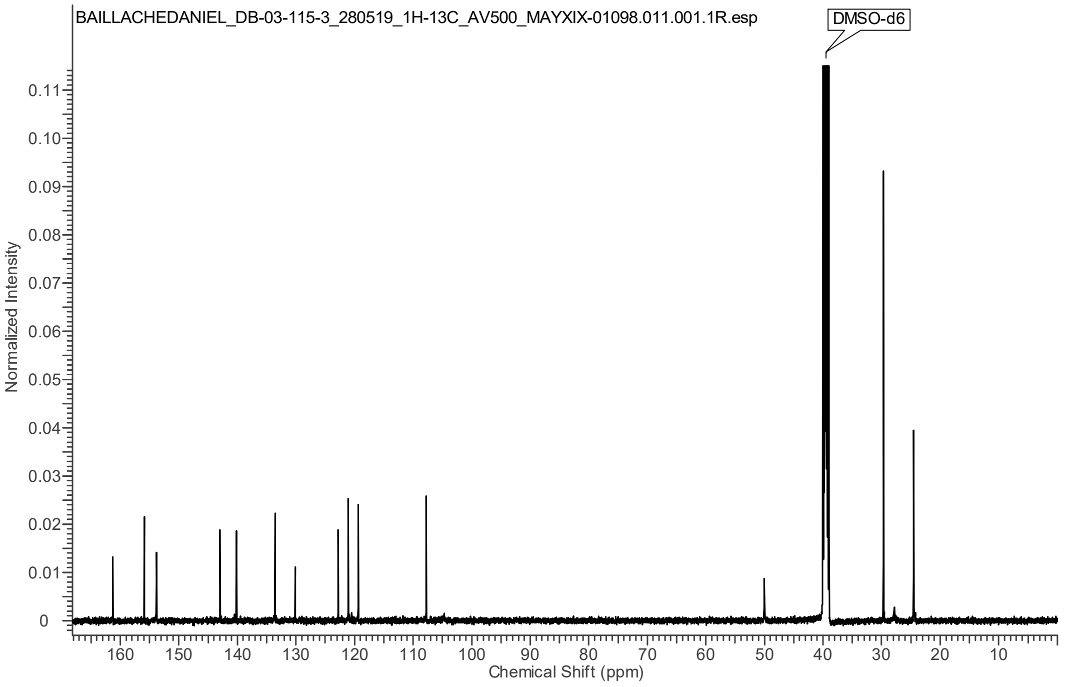
BAILLACHEDANIEL_DB-03-115-3_280519_1H-13C_AV500_MAYXIX-01098.011.001.1R.esp
DMSO-d6
Normalized Intensity
Chemical Shift (ppm)
